# The Cohesin Ring Uses Its Hinge to Organize DNA Using Non-topological as well as Topological Mechanisms

**DOI:** 10.1016/j.cell.2018.04.015

**Published:** 2018-05-31

**Authors:** Madhusudhan Srinivasan, Johanna C. Scheinost, Naomi J. Petela, Thomas G. Gligoris, Maria Wissler, Sugako Ogushi, James E. Collier, Menelaos Voulgaris, Alexander Kurze, Kok-Lung Chan, Bin Hu, Vincenzo Costanzo, Kim A. Nasmyth

**Affiliations:** 1 Department of Biochemistry, University of Oxford, South Parks Road, Oxford OX1 3QU, UK; 2 DNA Metabolism Laboratory, IFOM, The FIRC Institute of Molecular Oncology, Via Adamello 16, 21139 Milan, Italy; 3 Genome Centre, University of Sussex, Sussex House, Brighton BN1 9RH, UK; 4 Department of Molecular Biology and Biotechnology, University of Sheffield, Firth Court, Western Bank, Sheffield S10 2TN, UK

**Keywords:** loop extrusion, sister chromatid cohesion, cohesin, condensin, SMC, chromosome condensation

## Abstract

As predicted by the notion that sister chromatid cohesion is mediated by entrapment of sister DNAs inside cohesin rings, there is perfect correlation between co-entrapment of circular minichromosomes and sister chromatid cohesion. In most cells where cohesin loads without conferring cohesion, it does so by entrapment of individual DNAs. However, cohesin with a hinge domain whose positively charged lumen is neutralized loads and moves along chromatin despite failing to entrap DNAs. Thus, cohesin engages chromatin in non-topological, as well as topological, manners. Since hinge mutations, but not Smc-kleisin fusions, abolish entrapment, DNAs may enter cohesin rings through hinge opening. Mutation of three highly conserved lysine residues inside the Smc1 moiety of Smc1/3 hinges abolishes all loading without affecting cohesin’s recruitment to *CEN* loading sites or its ability to hydrolyze ATP. We suggest that loading and translocation are mediated by conformational changes in cohesin’s hinge driven by cycles of ATP hydrolysis.

## Highlights

• Chromatid cohesion is mediated by co-entrapment of sister DNAs inside cohesin rings
• Cohesin engages chromatin in non-topological as well as topological manners
• Cohesin loads onto chromatin despite closure of any of the three ring interfaces.
• Cohesin’s hinge domain is critical for non-topological and topological DNA association.

## Graphical Abstract

**Figure:**
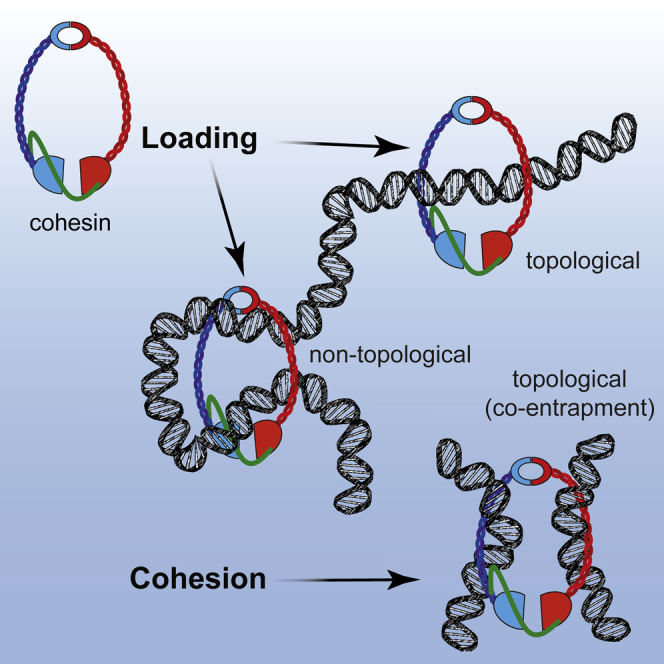

## Teaser

Cohesin can selectively engage DNA without entrapping it, suggesting a new dimension to cohesin function.

## Introduction

Smc/kleisin complexes facilitate chromosome segregation in bacteria as well as eukaryotes (Schleiffer et al., 2003). The latter have three types: condensin, cohesin, and the Smc5/6 complex (Haering and Gruber, 2016). Though initially identified as being essential for holding the sister chromatids together, cohesin shares with condensin an ability to organize DNA into chromatids. While condensin does this during mitosis (Hirano et al., 1997), cohesin does so during interphase (Tedeschi et al., 2013). It has been suggested that both types of complexes capture small loops of DNA and then extrude them in a processive manner (Nasmyth, 2001), a concept that has recently been embellished to explain the pattern of intra-chromosomal interactions during interphase as well as the process by which interactions between enhancers and distant promoters are regulated by the insulation factor CTCF (Fudenberg et al., 2016, Sanborn et al., 2015).

At cohesin’s core is a heterotrimeric ring containing a pair of SMC proteins, Smc1 and Smc3, and an α-kleisin subunit Scc1. Smc1 and Smc3 are rod-shaped proteins containing 50-nm-long intra-molecular anti-parallel coiled coils with a hinge/dimerization domain at one end and an ABC-like ATPase head domain composed of the protein’s N- and C-terminal sequences at the other. They bind each other via their hinges to form V-shaped heterodimers whose apical ATPases are interconnected by a single Scc1 polypeptide (Gruber et al., 2003). Scc1’s N-terminal domain (NTD) forms a four helical bundle with the coiled coil emerging from Smc3’s ATPase (Gligoris et al., 2014), while its winged helical C-terminal domain (CTD) binds to the base of Smc1’s ATPase (Haering et al., 2004). Bacterial Smc/kleisin complexes form similar structures, suggesting that asymmetric ring formation is a universal feature (Bürmann et al., 2013).

This structure raises the possibility that Smc/kleisin complexes associate with chromosomal DNAs by entrapping them inside their rings (Haering et al., 2008). Several types of such topological engagement have been envisaged for cohesin. Entrapment either of individual DNAs or loops of DNA might constitute the mechanism by which it associates with chromatin per se. On the other hand, sister chromatid cohesion could be conferred either by co-entrapment of sister DNAs inside the same ring (ring model) or through an association between two rings, each topologically engaged with DNA (handcuff model). Hitherto only entrapment of individual DNAs or of a pair of sister DNAs inside what appears to be a single cohesin ring has proven amenable to detection (Gligoris et al., 2014, Haering et al., 2008).

If cohesin associates with chromatin by entrapping DNA, then loading and release must involve passage of DNA through entry and exit gates, respectively. More is known about the mechanism of release. Cohesin dissociates from chromosomes after cleavage of Scc1 by separase at the onset of anaphase (Uhlmann et al., 1999) but at other stages of the cycle in a manner involving dissociation of the Smc3/Scc1 interface (Beckouët et al., 2016). This separase-independent releasing activity depends on a protein called Wapl (Kueng et al., 2006) that binds to a pair of hook-shaped proteins, associated with Scc1, namely, Scc3 and Pds5. Release also depends on a pair of highly conserved lysine residues (K112 and K113) on Smc3’s ATPase, whose modification by the acetyl transferase Eco1 during S phase abolishes release (Beckouët et al., 2016, Unal et al., 2008), thereby helping to maintain cohesion until the onset of anaphase.

The mechanism by which cohesin loads onto chromosomes is less well understood. It requires engagement of Smc1 and Smc3 ATPase heads as well as subsequent ATP hydrolysis (Arumugam et al., 2003). Neither Pds5 nor Wapl are necessary (Petela et al., 2017) but instead a separate complex containing Scc4 bound to the NTD of another Hawk protein called Scc2 is essential (Ciosk et al., 2000, Hu et al., 2015). Unlike release, which is blocked by fusion of Smc3’s ATPase to Scc1’s NTD, loading is not abolished by fusion of Smc3 or Smc1 ATPases to Scc1’s NTD or CTD, respectively, a finding that has led to the suggestion that DNAs enter via cohesin’s Smc1/Smc3 hinge domain (Gruber et al., 2006). Individual hinge domains have crescent shapes, and their interaction creates a pseudo-symmetric torus whose small lumen is invariably positively charged, even in fully symmetric bacterial hinges (Kurze et al., 2011).

In this study, we addressed the nature of cohesin’s association with DNA in cells at different stages of the cell cycle or with a variety of mutations that affect loading and/or cohesion. We found that co-entrapment of sister DNAs within cohesin rings invariably accompanies sister chromatid cohesion. On the contrary, although entrapment of individual DNAs normally accompanies loading, we describe a situation where this does not apply, namely, a cohesin mutant with a hinge whose positively charged lumen has been neutralized by five mutations (*smc1DDsmc3AAA*). The anomalous behavior of this hinge mutant implies that cohesin is able to load onto and move along chromosomes without associating with them in a topological manner.

During the course of mutating the inner surface of the hinge’s lumen, we discovered a triple mutation, replacing by aspartic acid three highly conserved lysines in Smc1, that greatly reduces cohesin’s association with chromosomes despite associating with Scc2 at *CEN* loading sites and being fully active as an ATPase. The behavior of this *smc1DDD* mutation implies that changes in the conformation of cohesin’s hinge that normally accompany ATP hydrolysis are essential for completion of the loading reaction as well as DNA entrapment. We suggest that both topological and non-topological modes of chromatin association depend on changes in cohesin’s Smc1/3 hinge domain that respond to changes in the state of its ATPase.

## Results

### Entrapment of Sister DNA Molecules by Hetero-trimeric Cohesin Rings

**Figure 1:**
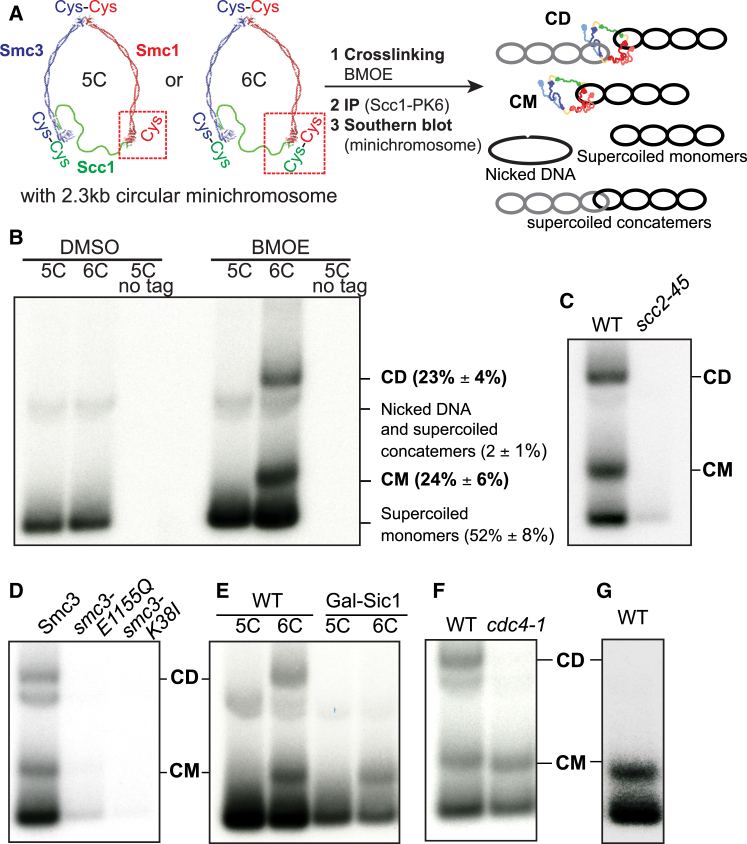
Entrapment of Single and Sister DNA Molecules by Hetero-trimeric Cohesin Rings (A) Procedure for detecting entrapment of DNAs by cohesin. 6C strains with cysteine pairs at all three ring subunit interfaces (2C Smc3: E570C S1043C, 2C Smc1: G22C K639C and 2C Scc1 C56 A547C) and 5C strains lacking just one of these cysteines (Scc1 A547C) and carrying a 2.3 kb circular minichromosome were treated with BMOE. DNAs associated with cohesin immunoprecipitates (Scc1-PK6) were denatured with SDS and separated by agarose gel electrophoresis. Southern blotting reveals supercoiled monomers and nicked and supercoiled concatemers along with two forms of DNA unique to 6C cells, termed CMs and CDs. (B) CMs and CDs in exponentially growing strains K23644 (5C), K23889 (6C), and K23890 (5C, no cohesin tag). Quantification of the bands (percentage of total) from the 6C crosslinked sample from 3 biological replicates is shown (data are represented as mean ± SD). See also Figure S1B. (C) CMs and CDs in WT (K23889) and *scc2-45* (K24267) 6C strains arrested in G1 with α factor at 25°C in YPD medium and released into nocodazole at 37°C. See also Figure S1D. (D) CM and CDs in exponentially growing 6C strains containing ectopically expressed versions of 2C Smc3-PK6: K24173 (WT Smc3), K24174 (smc3 E1155Q), and K24175 (smc3 K38I). (E) CMs and CDs in strains K23644 (5C), K23889 (6C), and those arrested in late G1 by expressing galactose-inducible nondegradable Sic1 K23971 (5C) and K23972 (6C). See also Figure S1E. (F) CMs and CDs in WT (K23889) and *cdc4-1* (K24087) 6C strains arrested in G1 at 25°C in YPD medium and released into YPD medium containing nocodazole at 37°C. See also Figure S1F. (G) CMs in α factor-arrested cells expressing non-cleavable 2C Scc1 (K24695). See also Figure S1G. See also Figure S1.

**Figure S1:**
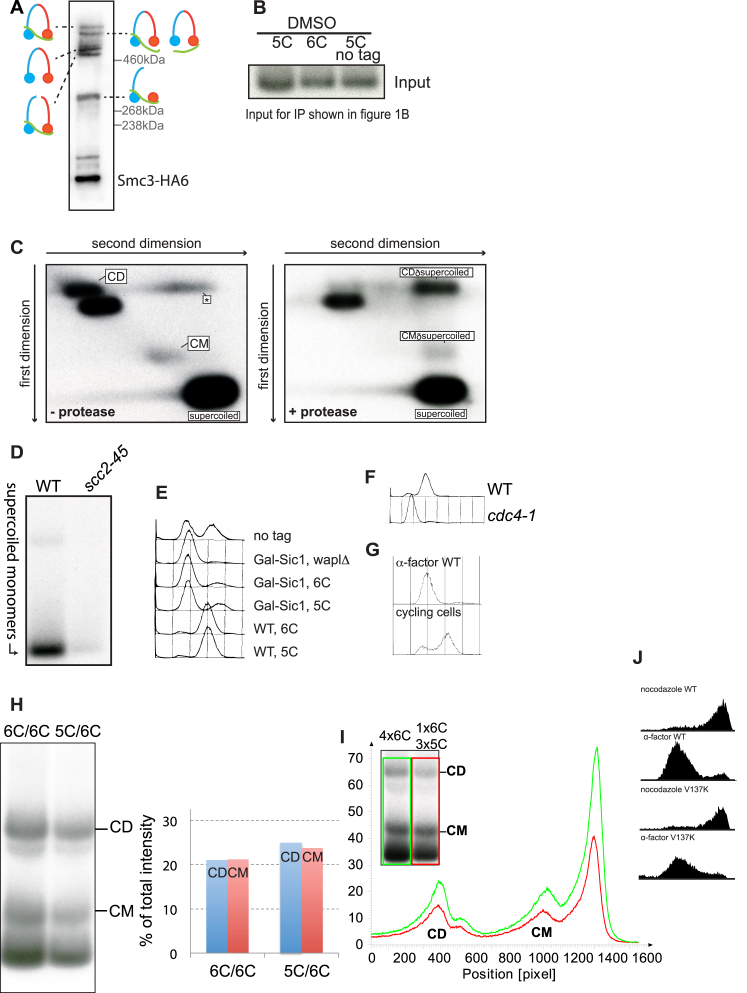
Related to Figures 1 and 2 (A) Western blot of fully circularizable 6C wild-type cohesin crosslinked *in vivo* using BMOE, probed for the HA-epitope on Smc3. The positions of the different crosslinked species are indicated. (B) Genomic DNA isolated from aliquots of the experiment in Figure 1B were electrophoresed, Southern blotted and detected with the TRP1 probe. (C) The 6C strain (K23889) was grown as in Figure 1B and subjected to 2D gel electrophoresis either in the absence or presence of proteinase K in the second dimension. The positions of the supercoiled monomer, CM and CD species are marked. ^(^^∗^^)^ indicates non-specific background signal. (D) Samples from experiment shown in Figure 1C were subjected to minichromosome IP without *in vivo* crosslinking; the position of the band containing supercoiled monomers is indicated in the Southern blot. (E) FACS profiles of the strains described in Figure 1E. (F) FACS profiles of the strains described in Figure 1F. (G) FACS profiles of the strain described in Figure 1G. (H) Exponentially growing diploid cells containing 2 copies of 6C cohesin with a tag on just one of the 2C Scc1 copies (K24242) and diploid cells containing 1 copy of 5C cohesin and one copy of 6C cohesin with a tag on just the 2C Scc1 (K24194) were subjected to the minichromosome IP assay. The intensities of the CM and CD bands quantified using AIDA Image Analyzer software are plotted as % of the total lane intensities. See also Figure 2E. (I) Quantification of the gel from Figure 2E showing the lane traces. (J) FACS profiles of the strains described in Figure 2G.

To measure DNA entrapment by cohesin, we created a pair of strains containing 2.3 kb circular minichromosomes: a 6C strain with cysteine pairs at all three ring subunit interfaces (Smc1G22C K639C, Smc3E570C S1043C, Scc1A547C C56) and a 5C strain lacking just one of these (Scc1A547C) (Figure 1A). Exponentially growing cells were treated with the cysteine-reactive homobifunctional crosslinker bismaleimidoethane (BMOE), which circularizes 20%–25% of 6C cohesin rings (Figure S1A) (Gligoris et al., 2014) and DNAs associated with cohesin immunoprecipitates separated by agarose gel electrophoresis following SDS denaturation. Southern blotting revealed two forms of DNA unique to 6C cells: one that migrates slightly slower than monomeric supercoiled DNAs (CMs) and a second that migrates slower than DNA-DNA concatemers (CDs) (Figures 1A and 1B). Little if any minichromosome DNA is detected in cells lacking the affinity tag on cohesin (Figure 1B). Importantly, electrophoresis in a second dimension following proteinase K treatment confirmed that both forms consist of monomeric supercoiled DNAs: CMs are single DNA molecules trapped within cohesin rings, while CDs contain a pair of sister DNAs trapped within tripartite cohesin rings (Figure S1C).

To address whether CMs and CDs correspond to loading and cohesion, respectively, we measured CM and CD formation in a variety of mutants and cell-cycle states. We first asked whether mutants defective in loading fail to create CMs and CDs. Accordingly, *scc2-45* cells failed to co-precipitate minichromosomes after undergoing DNA replication in the absence of functional Scc2 loader (Figure 1C). Likewise, a version of 6C cohesin that can bind but not hydrolyze ATP (Smc3E1155Q) and a version that cannot even bind ATP (Smc3K38I) failed to co-precipitate minichromosomes (Figure 1D). The formation of CMs and CDs therefore depends on both Scc2 and ATP hydrolysis. It is important to note that Smc3E1155Q cohesin associates with centromeres (*CEN*s) with a very high efficiency, whether measured by live imaging (Hu et al., 2011) or by calibrated chromatin immunoprecipitation sequencing (ChIP-seq) (Hu et al., 2015), and yet it largely fails to immunoprecipitate *CEN*-containing minichromosome DNAs.

### Cohesin Entraps Individual DNAs before DNA Replication

If cohesion were mediated by co-entrapment of sister DNAs within cohesin rings, then CDs should be detected only in cells that have undergone DNA replication. Likewise, if cohesin loading involved entrapment of individual DNAs, then CMs should be detected in cells that load cohesin onto chromosome prior to replication. Expression of a non-degradable version of the Cdk1 inhibitor Sic1 or inactivation of the F-box protein Cdc4, which is necessary for Sic1 degradation, prevents cells from entering S phase. In both cases, the failure to degrade Sic1 was accompanied by CM but not CD formation (Figures 1E and 1F). Expression of a version of Scc1A547C C56 that cannot be cleaved by separase in α factor-arrested G1 cells also led to formation of CMs but not CDs (Figure 1G). Thus, DNA replication is required for CDs but not for CMs and the latter are not merely a byproduct of CDs.

### Sister Chromatid Cohesion Is Accompanied by Entrapment of Sister DNAs within Individual Cohesin Rings

**Figure 2:**
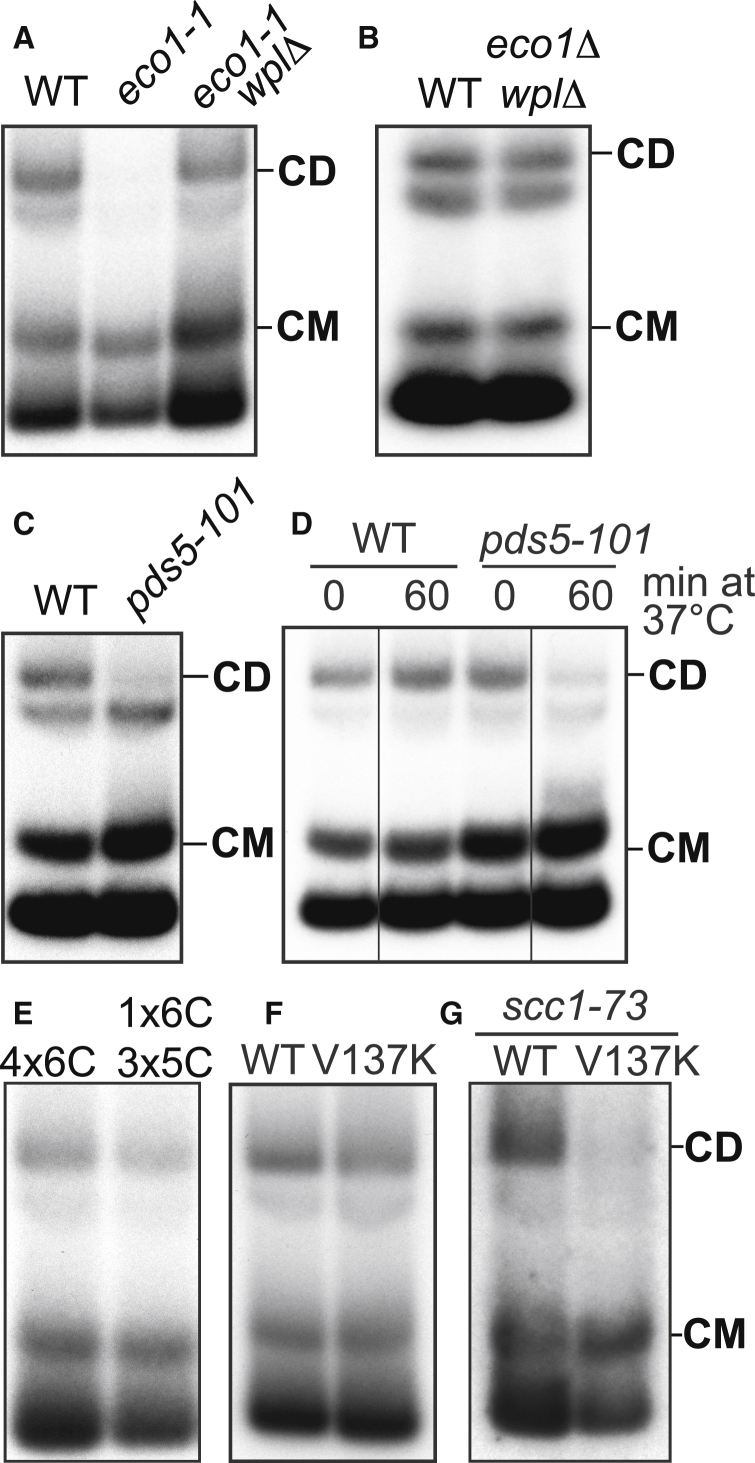
Sister Chromatid Cohesion Is Generated by Entrapment of Sister DNAs within Individual Cohesin Rings (A) CMs and CDs in WT (K23889), *eco1-1* (K23579), and *eco1-1 wplΔ* (K23578) strains arrested in G1 at 25°C in YPD medium and released into YPD medium containing nocodazole at 37°C. (B) CMs and CDs in exponentially growing WT (K23889) and *eco1Δ wplΔ* (Κ25287) 6C strains. (C) CMs and CDs in WT (K23889) and *pds5-101* (K24030) 6C strains arrested in G1 at 25°C in YPD medium and released into YPD medium containing nocodazole at 37°C. (D) CMs and CDs in exponentially growing WT (K23889) and *pds5-101* (K24030) 6C strains arrested in G2 by addition of nocodazole at 25°C and shifted to 37°C. Data are shown from the same Southern blot, with irrelevant lanes removed. (E) CMs and CDs in exponentially growing tetraploid cells containing 4 copies of 6C cohesin with a tag on just one of the 2C Scc1 copies (K24561) and tetraploid cells containing 3 copies of 5C cohesin and one copy of 6C cohesin with a tag on just the 2C Scc1 (K24560). See also Figures S1H and S1I. (F) CMs and CDs in exponentially growing 6C strains containing ectopically expressed versions of 2C Scc1, K24205 (WT), and K26413 (V137K) arrested in G2/M with nocodazole. (G) CMs and CDs in 6C strains with ts *scc1-73* allele at the endogenous locus and either WT (2C) Scc1 (K26600) or (2C) Scc1 V137K mutant (K26591) at an ectopic locus. Cells were arrested in G1 at 25°C in YPD medium and released into YPD medium containing nocodazole at 37°C. See also Figure S1J. See also Figure S1.

Though necessary, DNA replication is insufficient for CD formation. Thus, CDs fail to form in ts *eco1-1* cells when they undergo S phase at 37°C (Figure 2A). Loading is known to take place with high efficiency in such cells despite their failure to create stable cohesion (Chan et al., 2013), and indeed CM formation was unaffected (Figure 2A). The lack of CDs in *eco1* mutants is due to their failure to suppress releasing activity because *wpl1Δ* restores efficient CD formation (Figures 2A and 2B). The correlation between CDs and cohesion was strengthened by analysis of pds5 mutants. While essential for maintaining cohesion, Pds5 is not required for loading (Panizza et al., 2000, Petela et al., 2017). Accordingly, *pds5-101* cells that undergo DNA replication at 37°C form CMs but no CDs (Figure 2C). Interestingly, CM accumulation was marginally elevated in *pds5-101* cells, possibly because of reduced releasing activity (Figure 2C).

It has been suggested that loss of cohesion caused by inactivation of Pds5 during G2/M despite persistence of cohesin on chromosomes is evidence that cohesin cannot hold sister DNAs together by entrapping them within a single cohesin ring (Tong and Skibbens, 2014). To address this, we arrested 6C *pds5-101* cells in G2/M at the permissive temperature and then shifted the cells to the restrictive temperature, which is known to destroy cohesion (Panizza et al., 2000). This led to a reduction of CDs (by 70%; 3 biological replicates) but not CMs (Figure 2D). Thus, loss of cohesion during G2/M in *pds5* mutants is accompanied by loss of CDs, extending yet again the correlation between cohesion and CDs. The persistence of cohesin on chromosomes in *pds5* mutants does not therefore refute the notion that cohesion is mediated by CDs.

To address in a more definitive fashion whether individual hetero-trimeric rings or versions containing two or more kleisin subunits hold CDs together, we created two tetraploid strains that either contain four copies of covalently circularizable cohesin (4×6C), or three copies of 5C (lacking 1 of the 6 cysteine residues needed for complete circularization) and one copy of 6C cohesin. If individual cohesin rings held CDs together, the ratio of CDs to CMs should be unaltered by any reduction in the fraction of circularizable cohesin. However, if CDs were mediated by oligomeric cohesin containing two (or more) Scc1 subunits then the fraction should be one quarter (or less) in the case of 6C/5C/5C/5C tetraploids, compared to 4×6C controls. In fact, the ratio of CDs to CMs in both these strains was very similar in 3 biological replicates (Figure 2E; $\left({\left(CD/CM\right)}^{4x6C}/{\left(CD/CM\right)}^{1x6C,3x5C}\right)=1.01,\;\text{SD}=\text{0}\text{.}100$). The same was true in diploid cells analyzed in a similar fashion (Figure S1H), implying that rings containing only a single copy of Scc1 hold the two DNAs within CDs together.

### Cohesin Rings Collaborate to Form CDs

In the course of exploring the relationship between CDs and cohesion, we analyzed cells expressing an *scc1* mutant (*V137K*) that is defective in binding Pds5 (Chan et al., 2013) and therefore lethal. To do this, we constructed a 6C *V137K* mutant strain that was kept alive by an untagged copy of the endogenous *SCC1* gene. Due to its inability to recruit Pds5, we expected that cohesin containing Scc1V137K would be able to form CMs but not CDs. To our surprise, *scc1V137K* caused only a slight if any reduction in CDs (Figure 2F). This could be either because CD formation is insufficient for cohesion, or wild-type cohesin, which cannot actually be part of the CDs associated with V137K cohesin, facilitates formation of CDs by the mutant complexes. To test the latter possibility, we replaced the endogenous wild-type *SCC1* with the temperature-sensitive *scc1-73* allele and measured whether V137K is still capable of forming CDs when cells underwent S phase at the restrictive temperature. Under these conditions, *scc1V137K* supported CM but not CD formation (Figure 2G). We conclude that wild-type cohesin helps CD formation and/or maintenance by V137K cohesin.

Complementation between mutant *scc1* alleles had previously indicated that different cohesin complexes might interact functionally (Eng et al., 2015), and this observation was cited as evidence that cohesion is instead mediated by a pair of cohesin complexes. Our demonstration that wild-type Scc1 enables Scc1V137K to form CDs provides an alternate interpretation; namely, that two or more cohesin rings collaborate to produce cohesive structures that contain sister DNAs held within individual rings.

### DNA Entrapment Is Necessary for Cohesion, but Not for Loading or Translocation

**Figure 3:**
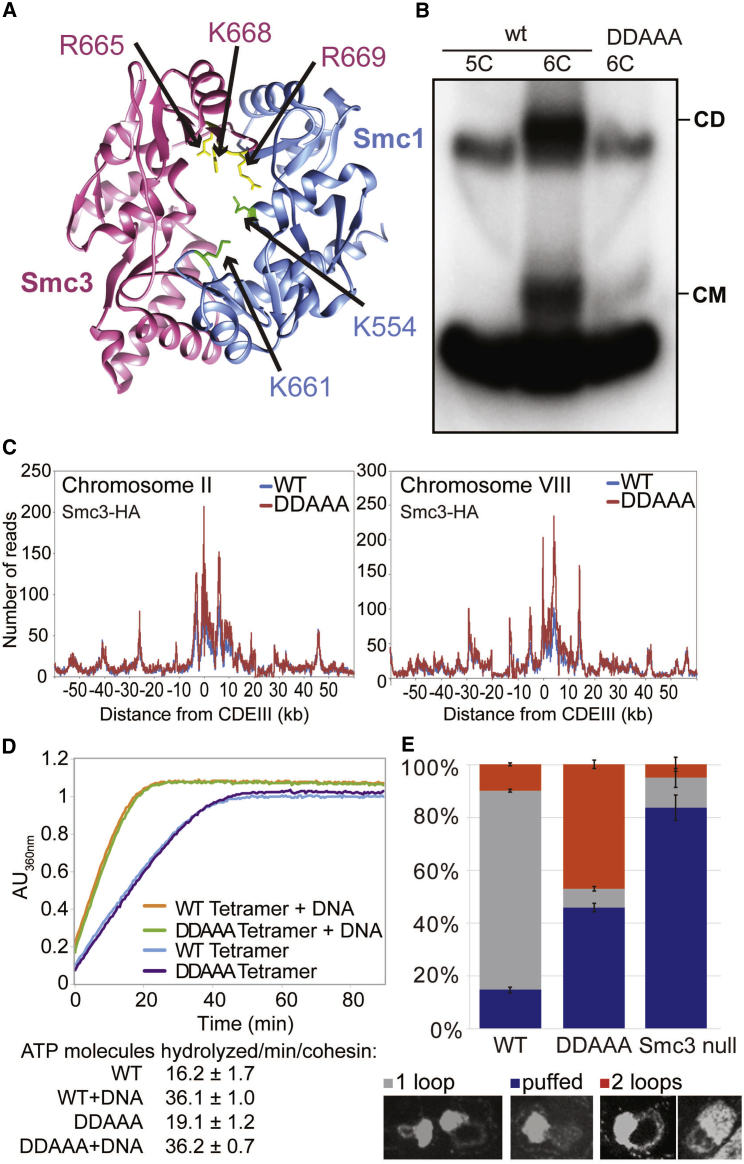
DNA Entrapment Is Necessary for Cohesion but Not for Loading or Translocation (A) Structure of the mouse hinge domain, highlighting positively charged residues in its central channel neutralized by *smc1K554D K661D smc3R665A K668A R669A* mutations (DDAAA). (B) CMs and CDs in exponentially growing K23644 (5C) and two 6C strains (K26210 with an ectopic WT 2C *SMC1* and K26215 with endogenous 2C *smc3AAA* and ectopic 2C *smc1DD* (*DDAAA*)). Over three biological replicates, the intensities of CM and CD bands in the DDAAA mutant were reduced to around 20% and 3% of the WT levels, respectively. (C) Exponentially growing strains WT (K15426, Smc3-HA) and DDAAA mutant (K15424, smc3AAA-HA) were analyzed by calibrated ChIP-sequencing. ChIP profiles along chromosomes II and VIII are shown. See also Figure S2C. (D) ATPase activity of purified WT and DDAAA mutant tetramer stimulated by Scc2. The rate of ATP hydrolysis was measured either in the presence or absence of DNA. (E) Strains K26797 (containing endogenous 3× miniAID-tagged *SMC3* and ectopic WT *SMC3*), K26611 (containing endogenous 3× miniAID tagged-*SMC3* and endogenous *smc1DD* and ectopic *smc3AAA*), and K26767 (3× miniAID-tagged *SMC3* and no ectopic *SMC3*) were arrested in G1 and synthetic auxin (indole-3-acetic acid) added to 1 mM 30 min before release. Cultures were released into YPD containing 1 mM auxin and nocodazole. 60 min after release from the G1 arrest, cultures were harvested and chromosomes spread (STAR Methods). Micrographs of chromosome masses of the two strains were quantified from three independent experiments (n = 100) and categorized as “1 loop” (showing fully condensed rDNA loops), “2 loops” (showing fully condensed rDNA loops that are split because of loss of cohesion), and “puffed” (showing unstructured, puffed rDNA morphology). See also Figure S4. Data are represented as mean ± SD. See also Figures S2, S3, and S4.

**Figure S2:**
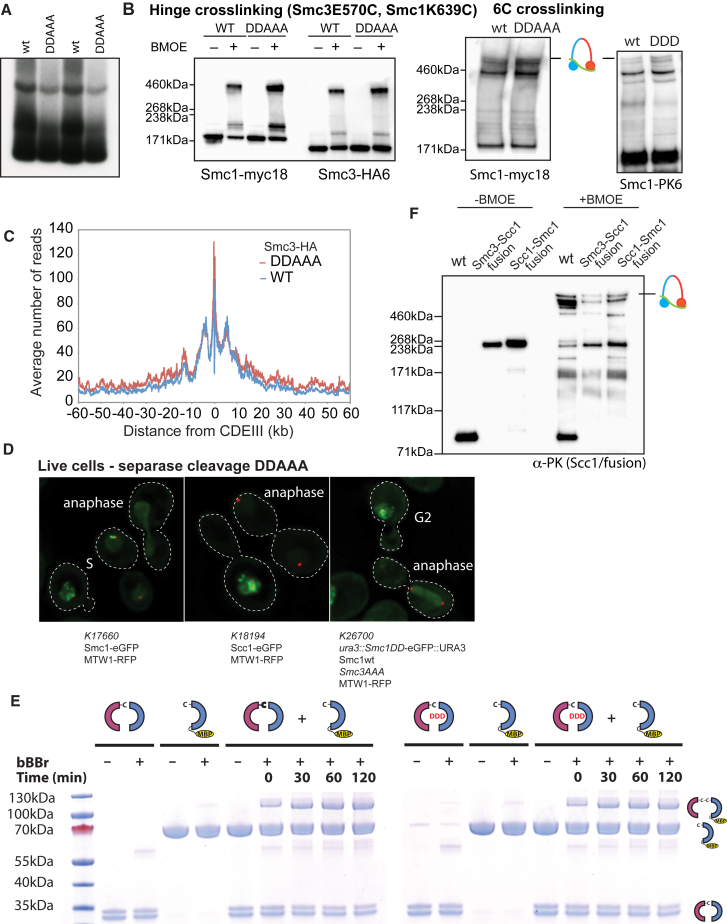
Related to Figures 3, 4, 5, and 6 (A) Two replicates of the experiment performed in Figure 3B. (B) Left panel: Efficiency of the hinge crosslinking was compared between the wild-type and the DDAAA strains: Wild-type (K26085) and DDAAA (K26086) strains were subjected to western blotting with and without *in vivo* crosslinking and the blots probed with anti-Myc (Smc1) and anti-HA (Smc3) antibodies to detect the un-crosslinked and crosslinked Smc1/Smc3 species. Right panels: Samples from experiment described in Figures 3B and 4F were subjected to western blotting after *in vivo* crosslinking and the blots probed with either anti-Myc (left panel) or anti-PK antibody (right panel). The positions of the fully circularized species are indicated. (C) Average ChIP profiles of the experiment described in Figure 3C. The ChIP profiles are showing the number of reads at each base pair away from the CDEIII element averaged over all 16 chromosomes. (D) Exponentially growing diploid strains K17660 (expressing Mtw1-RFPand Smc1-eGFP), K18194 (expressing Mtw1-RFP and Scc1-eGFP), and K26700 (expressing Mtw1-RFP and with smc3AAA expressed from the endogenous locus and expressing smc1DD from an ectopic locus) were grown in YEPD medium at 25°C and were placed on 2.5% agarose pads made of synthetic complete medium containing glucose. Live cell imaging was performed under a spinning disk confocal system at 25°C. (E) Coomassie stained gels showing Smc1/3 hinge exchange. Purified heterodimeric hinge domains that had either wild-type or DDD mutant Smc1 associated with Smc3 hinge containing a cysteine substation (E570C) were mixed with purified MBP-tagged Smc1 containing a cysteine substitution (K639C). We measured the exchange of the wild-type and DDD mutant Smc1 with the MBP-Smc1 by adding homo-bifunctional crosslinker bBBr for the indicated times. (F) Crosslinking was compared between the wild-type, Smc3-Scc1 and Scc1-Smc1 fusion strains: Wild-type (K23889), Smc3-Scc1 (K24838) and Scc1-Smc1 (K25696) strains were subjected to western blotting with and without *in vivo* crosslinking and the blots probed with anti-PK antibody against Scc1/fusion proteins to detect un-crosslinked and crosslinked species. The position of the fully circularized species is indicated.

**Figure S3:**
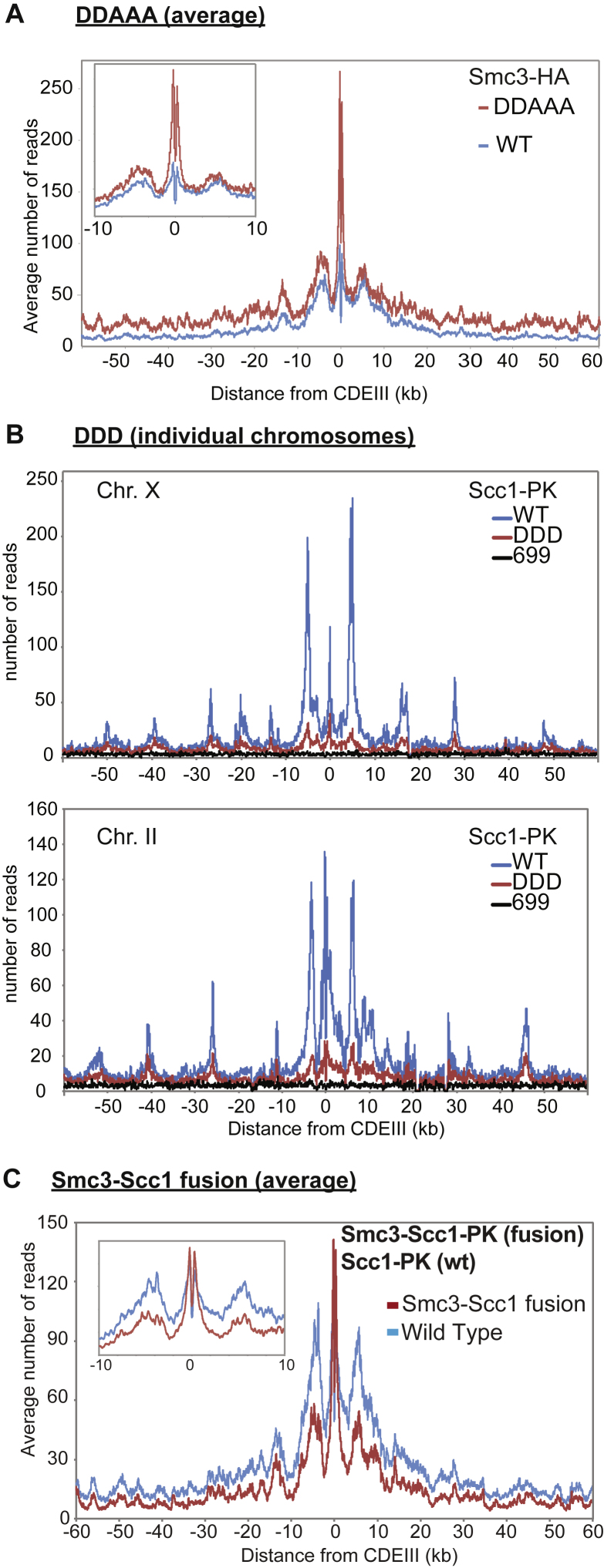
Related to Figures 3, 4, and 6 (A) Cells from K26797 (containing endogenous 3×miniAID-tagged *SMC3* and ectopic wild-type *SMC3*), K26611 (containing endogenous 3×miniAID tagged-*SMC3* and endogenous *smc1DD* and ectopic *smc3AAA*) were arrested in G1 and synthetic auxin (indole-3-acetic acid) added to 1 mM 30 min before release. Cultures were released into YPD containing 1mM auxin and nocodazole to arrest the cultures in G2/M and analyzed by calibrated ChIP-sequencing. ChIP profiles show the number of reads at each base pair away from the CDEIII element averaged over all 16 chromosomes. (B) Calibrated ChIP-seq profiles along chromosome II and Chromosome X from the experiment described in Figure 4E. (C) Calibrated ChIP-seq of exponentially growing wild-type (K23889) and Smc3-Scc1 fusion strain (K24838). ChIP profiles show the number of reads at each base pair away from the CDEIII element averaged over all 16 chromosomes. See Figure 6B for representative individual chromosome traces.

In a final attempt to refute the notion that CMs and CDs reflect loading and cohesion, respectively, we analyzed a quintuple mutant that neutralizes the hinge lumen’s positive charge (Figure 3A). *smc1K554D K661Dsmc3R665A K668A R669A* (*smc1DDsmc3AAA*) cohesin loads onto and moves along chromosomes in a similar manner to wild-type but fails to generate cohesion and is only poorly acetylated by Eco1 (Kurze et al., 2011). As expected, a 6C version of *smc1DDsmc3AAA* failed to produce CDs. More surprising, it also largely failed to form CMs (reduced to approximately 20% of 6C wild-type (WT) levels in the fraction of immunoprecipitated DNA; Figures 3B and S2A) despite forming tripartite rings (Figure S2B). Crucially, the level of minichromsome DNAs in *smc1DDsmc3AAA* immunoprecipitates was similar to WT, showing that the mutant protein associates stably with chromatin, even though it does not entrap it (Figure 3B). Calibrated ChIP-seq confirmed this result: loading of *smc1DDsmc3AAA* cohesin throughout the genome was similar, if not greater, than that of WT cohesin both in the presence (Figure 3C) or absence (Figure S3A) of endogenous untagged WT complexes. Calibrated ChIP-seq also showed that mutant complexes loaded at *CEN*s migrate into neighboring sequences like WT resulting in similar distributions of the WT and mutant complexes (Figure 3C). Because loading and possibly also subsequent movement from loading sites require stimulation of cohesin’s ATPase activity by Scc2, we purified WT (*SMC1 SMC3 SCC1 SCC3*) and mutant (*smc1DD smc3AAA SCC1 SCC3)* tetramers and compared their ATPase activity in the presence of Scc2 and in the presence and absence of DNA. Crucially, *smc1DDsmc3AAA* had no effect on ATPase activity (Figure 3D).

Four important conclusions can be drawn from the behavior of *smc1DDsmc3AAA* cohesin. First, *smc1DDsmc3AAA* causes a highly specific defect in entrapping DNA. Remarkably, it causes this defect without affecting loading or in the case of centromeres migration away from *CEN* loading sites. Second, the Smc1/3 hinge must be intimately involved in the entrapment process. Third, because the entrapment defect is accompanied by a failure to build sister chromatid cohesion, entrapment is presumably necessary for cohesion. Last but not least, cohesin can load onto, move along, and remain stably associated with chromatin in the absence of DNA entrapment. Hitherto, topological entrapment has been the only explanation for loading as well as release through separase-mediated Scc1 cleavage or Wapl-mediated dissociation of the Smc3/Scc1 interface. We now know that, although entrapment clearly does take place, it cannot be the only mechanism of DNA association. Interestingly, the non-topological chromosomal *smc1DDsmc3AAA* cohesin is still removed by separase during anaphase (Figure S2D).

### Organization of DNA into Chromatid-like Threads Does Not Require Entrapment of DNA by Cohesin Rings

We next addressed whether *smc1DDsmc3AAA* cohesin is still active in organizing chromosome topology. The tandem array of rDNA repeats assembles into threads during M phase, albeit ones that are much thinner than those of conventional mitotic chromosome. Importantly, formation of these threads depends on cohesin (Guacci et al., 1994). Because thread formation is not dependent on sister chromatid cohesion in mammalian cells, it is possible that the same might be true of mitotic rDNA threads in yeast. If so, and if *smc1DDsmc3AAA* cohesin still possesses this thread-making activity, then post-replicative *smc1DDsmc3AAA* cells should contain not one but two rDNA threads.

**Figure S4:**
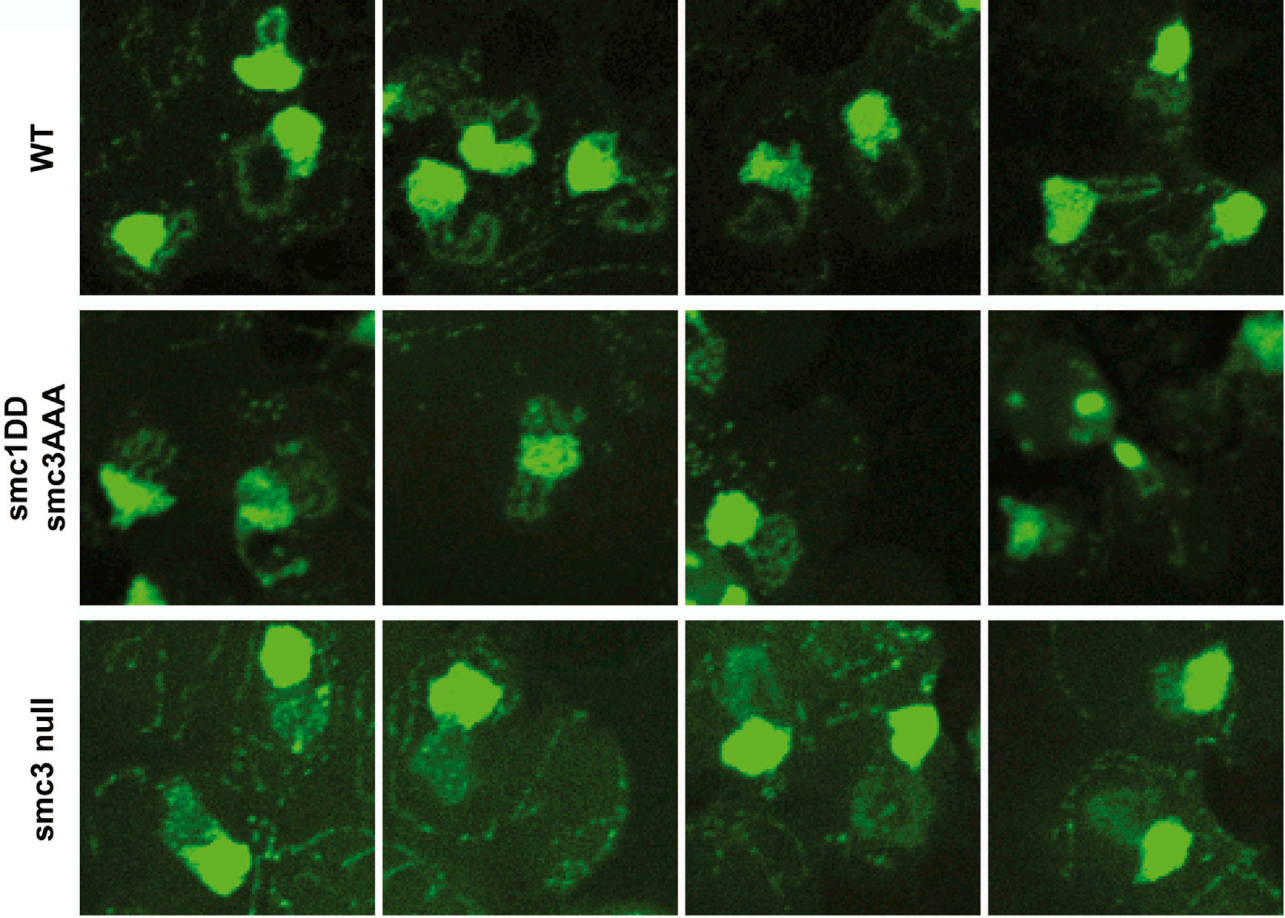
Related to Figure 3E Examples of chromosome spreads of the wild-type, DDAAA mutant and the smc3 depletion (smc3 null) strains from the experiment described in Figure 3E.

To test this, cells whose *SMC3* gene had been replaced by a 3× miniAID-tagged version (*smc3-AID*), expressing *smc1DD* from the endogenous locus and *smc3AAA* from an ectopic one were allowed to undergo DNA replication in the presence of auxin, which induces degradation of the AID-tagged endogenous Smc3 protein (Li et al., 2017). Their behavior was compared to *smc3-AID* cells with a WT *SMC1* gene and expressing *SMC3* from an ectopic locus (the WT control) as well as to cells lacking an ectopic *SMC3* gene (the *smc3* mutant control). As expected most WT cells contained a single rDNA thread, which forms a distinct loop connected to but separate from the rest of chromosome XII, which is situated within an amorphous mass of chromatin containing all 15 other chromosomes. Cells that had undergone S phase without Smc3 lacked discernable rDNA loops (Figures 3E and S4). Remarkably, cells that had undergone S phase expressing only *smc1DDsmc3AAA* cohesin frequently (47%) contained a pair of thin rDNA loops (Figures 3E and S4). This implies that *smc1DDsmc3AAA* cohesin can organize individual rDNA into threads but not hold sister rDNA threads together.

### Highly Conserved Lysine Residues inside Smc1/3 Hinges Are Required for All Types of Cohesin Loading

**Figure 4:**
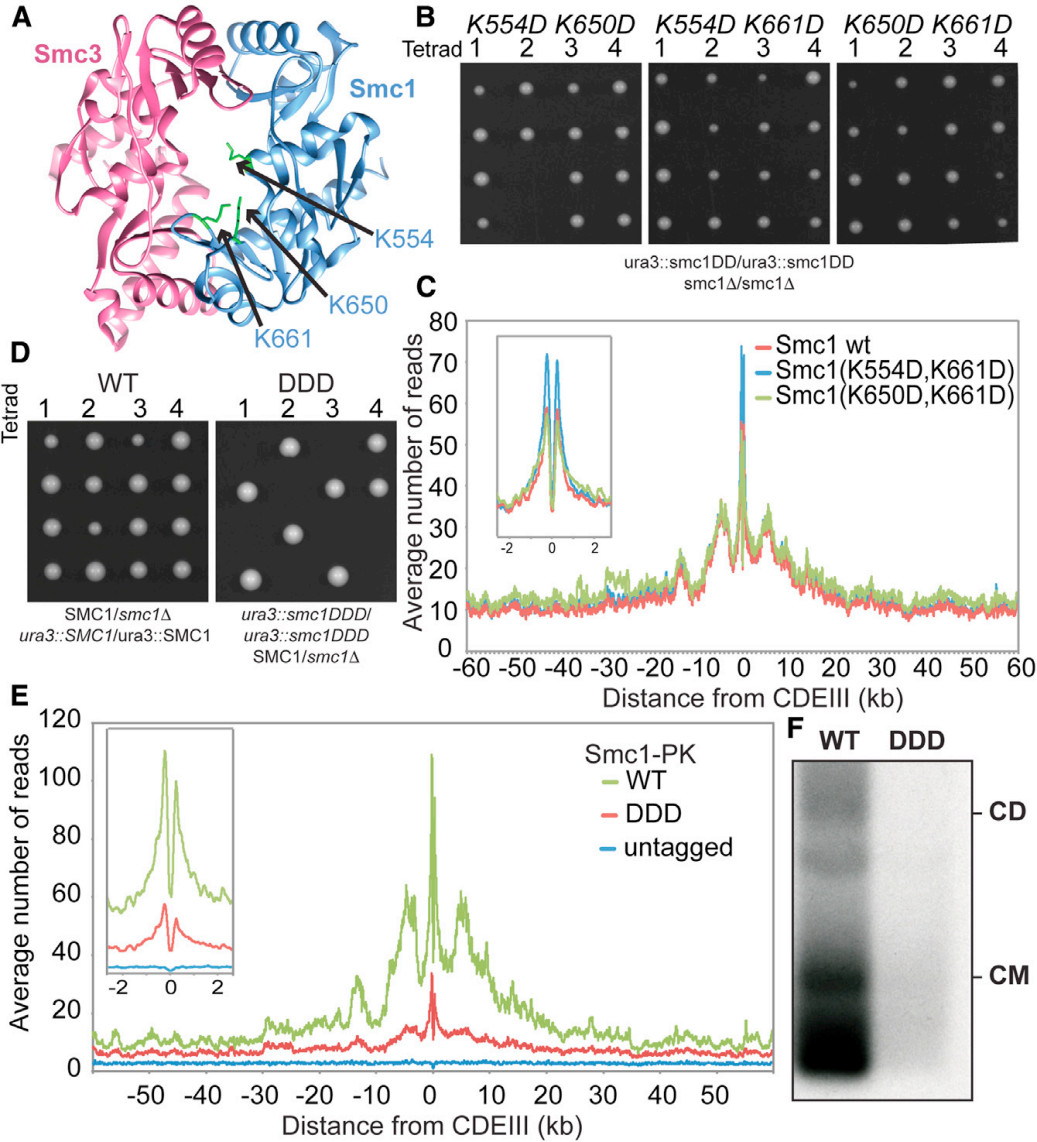
Residues within Its Hinge Domain Dictate Cohesin’s Ability to Load onto Chromosomes (A) Structure of the mouse hinge depicting mutated Smc1 residues (*smc1 K554D K650D K661D*) (DDD). (B) Haploid segregants following tetrad dissection of asci from diploid strains (*ura3::smc1DD /ura3::smc1DD smc1Δ/smc1Δ* containing two mutations of all possible combinations from K554D, K650D, and K661D). (C) Calibrated ChIP-seq of exponentially growing strains with a deletion of the endogenous *SMC1* gene and expressing ectopically either WT *SMC1* (K15324), *smc1K554D K661D* (K15322), or *smc1K650D K661D* mutant (K15226). (D) Haploid segregants following tetrad dissection of asci from diploid strains (SMC1/smc1Δ ura3::SMC1/ura3::SMC1) and (SMC1/smc1Δ ura3::smc1DDD/ura3::smc1DDD). (E) Calibrated ChIP-seq of exponentially growing strains K24327 (ectopic *SMC1*), K26756 (ectopic *smc1DDD*), and K699 (untagged control). (F) Minichromosome IP assay of exponentially growing strains K24327 (expressing ectopic WT 2C *SMC1*) and K26610 (expressing ectopic 2C *smc1DDD*). See also Figures S2, S3, S5, S6, and S7.

**Figure S5:**
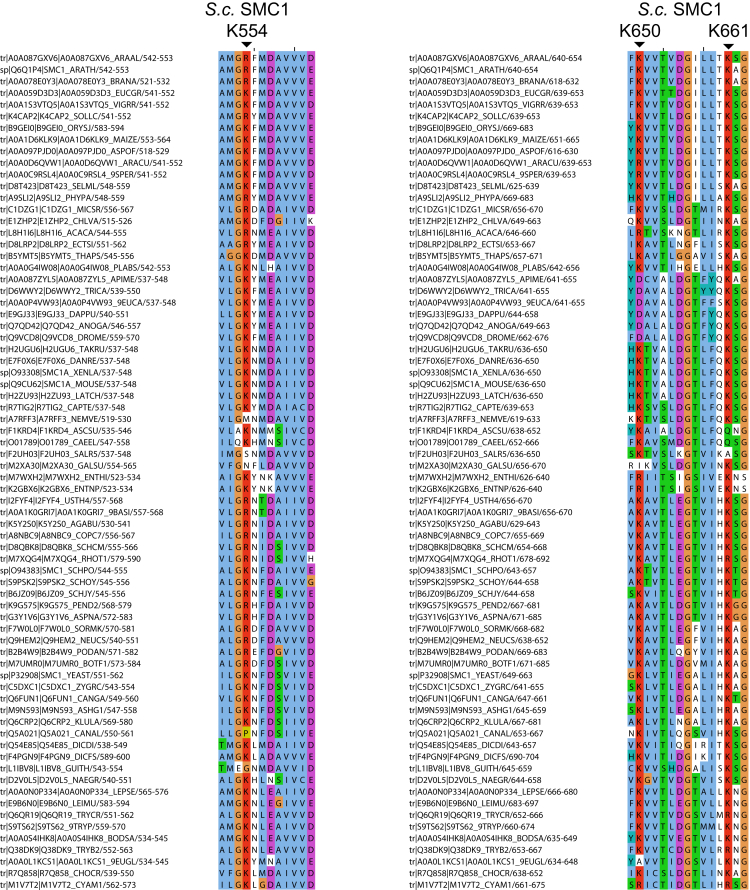
Related to Figures 4 and 5 Multiple sequence alignment indicating conservation of Smc1 resides K554, K650 and K661 in *S. cerevisiae* across various other eukaryotes. These residues are highlighted in the hinge structure shown in Figure 4A.

To address whether cohesin’s hinge might be involved in all aspects of cohesin’s chromosome organization and not merely the DNA entrapment intrinsic to sister chromatid cohesion, we undertook a more systematic analysis of the role of basic residues within cohesin’s hinge. Smc1K554 and K661 are in fact part of a triad of highly conserved lysine residues, including K650, residing within the Smc1 moiety of the hinge’s lumen (Figures 4A and S5). All double-mutant combinations involving lysine to aspartate substitutions, namely, *smc1K554D K650D*, *smc1K554D K661D*, and *smc1K650D K661D*, are viable (Figure 4B). Indeed, calibrated ChIP-seq showed that neither *smc1K554D K650D* nor *smc1K650D K661D* had any appreciable effect on cohesin’s association with the genome, either around centromeres or along chromosome arms (Figure 4C).

In contrast, the *smc1K554D K650D K661D* triple mutant (*smc1DDD*) was lethal (Figure 4D), greatly reduced cohesin’s association with chromatin throughout the genome (Figure 4E), and abolished co-precipitation of minichromosome DNA with cohesin as well as formation of CMs and CDs when present as a 6C version (Figure 4F). Treatment of cells with the 6C version of *smc1DDD* showed that chemical circularization of the triple mutant was identical to WT, demonstrating that the triple mutation does not adversely affect Smc1/3 hinge dimerization or indeed association between Smc1 and Smc3 ATPase domains with CTDs and NTDs of Scc1, respectively (Figure S2B). Because *smc1DDsmc3AAA* reduces the off-rate of isolated Smc1/3 hinge complexes (Kurze et al., 2011), a competition crosslinking assay was used to measure this property, which showed that *smc1DDD* had no effect (Figure S2E).

**Figure 5:**
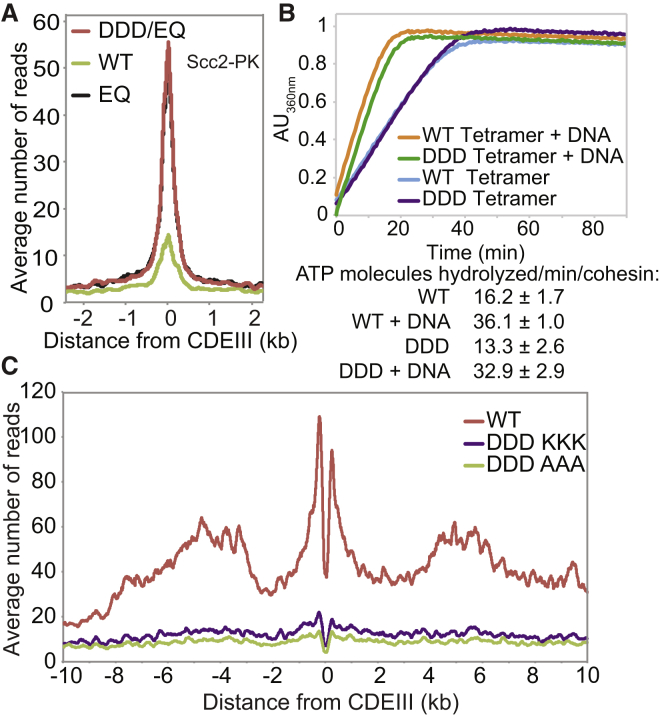
*smc1DDD* Mutation Does Not Affect Scc2-Stimulated ATP Hydrolysis Cycle (A) Calibrated ChIP-seq of exponentially growing strains each containing Scc2-PK6, K26839 (ectopic *SMC1*), K26840 (ectopic *smc1DDD E1158Q*), and K25646 (ectopic *smc1 E1158Q*). (B) ATPase activity of purified WT and DDD mutant tetramer stimulated by Scc2. ATP hydrolysis was measured with and without DNA. (C) Exponentially growing strains K26756 (expressing the WT Smc3 from the endogenous locus and the smc1DDD mutant from an ectopic locus), K26757 (expressing smc1 DDD mutant from an ectopic locus and smc3AAA mutant from the endogenous locus), and K24327 (expressing WT Smc1 and Smc3) were analyzed by calibrated ChIP sequencing (Smc1-PK IP). The ChIP profiles are showing the number of reads at each base pair away from the CDEIII element averaged over all 16 chromosomes. See also Figures S2, S5, and S7.

Analysis of mutations like *smc1E1158Q* and *smc3E1155Q* that block ATP hydrolysis has revealed two steps in the loading reaction at *CEN*s. The first is association with *CEN*s of cohesin whose ATPase heads have engaged in the presence of ATP while its kleisin subunit binds Scc2 instead of Pds5. A subsequent step involves conversion of this unstable intermediate into a complex that moves up to 30 kb into neighboring peri-centric sequences, while remaining stably associated with chromatin. Formation of the unstable Scc2-bound intermediate at *CEN*s can be detected using calibrated ChIP-seq by measuring enhancement by *smc1E1158Q* of Scc2’s association with *CEN*s (Petela et al., 2017). Importantly, the enhanced recruitment of Scc2 to *CEN*s in *smc1DDD smc1E1158Q*-expressing cells was identical to that in *smc1E1158Q*-expressing cells (Figure 5A), which suggests that *smc1DDD* affects the second and not the first step in the loading reaction. Because *smc1DDD* has no effect on ATPase activity induced by Scc2 *in vitro* (Figure 5B), we conclude that *smc1DDD* does not affect the ATP hydrolysis cycle per se but instead a change in cohesin’s conformation that normally accompanies hydrolysis of ATP bound to its ATPase heads, presumably involving the hinge and the associated coiled coils.

To address why *smc1DDsmc3AAA* merely blocks entrapment while *smc1DDD* hinders all types of loading including entrapment, we created an *smc1DDDsmc3AAA* sextuple mutant. Calibrated ChIP-seq revealed that *smc3AAA* cannot ameliorate *smc1DDD’s* loading defect (Figure 5C). In other words, *smc1K650D* is epistatic to *smc3AAA* in *smc1K554D K661D* cells. It is remarkable that mutation of any one of three conserved lysines is sufficient to reduce WT levels of loading in double-*smc1DD* mutants to lethally low levels. None of the three conserved lysines has a unique role and all make “equivalent” contributions. Positive charge per se and not precisely where it is situated within the hinge’s lumen appears to be crucial.

### Efficient Entrapment of DNAs when Smc and Kleisin Subunits Are Fused

If co-entrapment of sister DNAs mediates sister chromatid cohesion, then the cohesin ring must somehow open up, creating a gate through which DNAs can enter. A recent study suggested that an entry gate is created by transient dissociation of the Smc3/Scc1 interface (Murayama and Uhlmann, 2015). A problem with this claim is that it is inconsistent with the fact that Smc3-Scc1 and Scc1-Smc1 fusion proteins are functional (Gruber et al., 2006), casting doubt on either Smc-kleisin interface being an obligatory entry gate.

**Figure 6:**
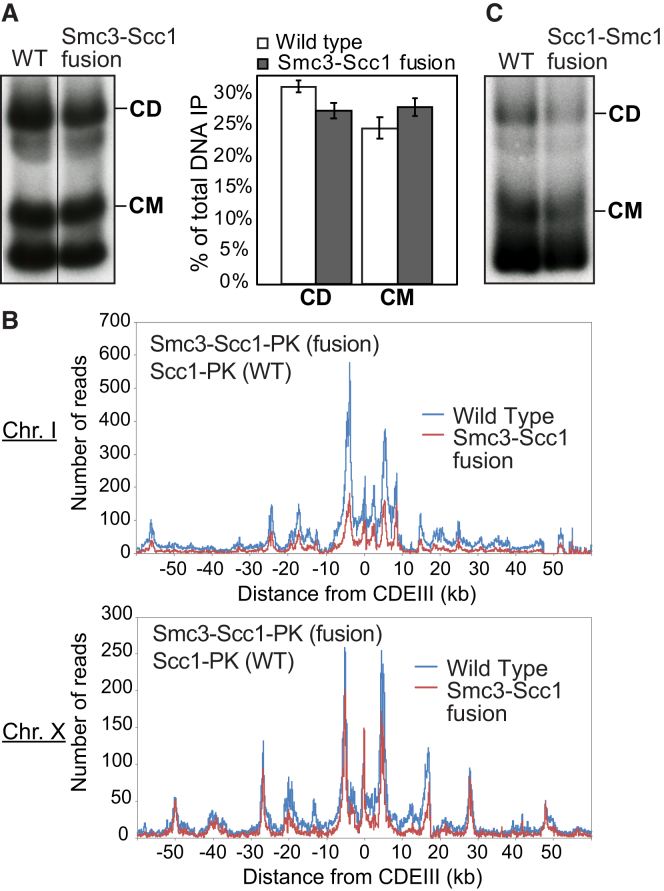
Entrapment of DNAs When Smc and Kleisin Subunits Are Fused Together (A) CMs and CDs in an exponentially growing 6C WT (K23889) strain and a strain containing 2C *SMC1* and expressing an *SMC3-SCC1* fusion containing cysteines in Smc3’s hinge and Scc1’s C terminus (K24838) as the sole source of Smc1 and Scc1. Data are shown from the same Southern blot, with one irrelevant lane removed. The fractions of CD and CM of the total DNA immunoprecipitates from WT and fusion strains across 3 biological replicates are shown (data are represented as mean ± SD). (B) Calibrated ChIP-seq of exponentially growing WT (K23889) and Smc3-Scc1 fusion strain (K24838). Calibrated ChIP-seq profiles of representative chromosomes I and X are shown. See Figure S3B for a representation of the average of all 16 chromosomes. (C) CMs and CDs in exponentially growing 6C WT (K23889) strain and a 4C strain (containing an Smc3-Scc1 fusion protein with cysteine pairs at the hinge and Scc1/Smc1 interfaces but not at the Smc3/Scc1 interface) expressing a PK3-Scc1-Smc1 fusion as the sole source of Scc1 and Smc1 (K25696). See also Figures S2 and S3.

It is known that Smc3-Scc1 fusion proteins are capable of forming CDs but not how efficiently (Haering et al., 2008). To address this, we compared the ability of WT 6C complexes to form CMs and CDs with that of 4C complexes containing an Smc3-Scc1 fusion protein with cysteine pairs at the hinge and Scc1/Smc1 interfaces but not at the Smc3/Scc1 interface. Smc3-Scc1 4C containing complexes were capable of forming CMs and CDs with a similar efficiency to that of WT 6C complexes (Figure 6A). Calibrated ChIP-seq showed that Smc3-Scc1 fusion proteins load at *CEN*s and move into peri-centric sequences in a manner similar to WT, albeit slight less efficiently (Figures 6B and S3B). Because crosslinked Smc3-Scc1 complexes do not form higher order multimers (Figure S2F), Smc3-Scc1 cohesin presumably entraps minichromosomes as a monomeric ring, as in WT.

Because neither CMs nor CDs would be possible in 4C Smc3-Scc1 cells if the linker connecting Smc3 and Scc1 were cleaved, these results confirm that the Smc3/Scc1 interface cannot be an obligatory entry gate for the DNA. They do not, however, exclude the possibility that loading can or indeed does take place via this interface. 4C Scc1-Smc1 strains behaved similarly, proving that DNAs can also enter rings without opening the Scc1/Smc1 interface (Figure 6C).

### Covalent Closure of Cohesin’s Hinge Interface Fails to Block Loading in *Xenopus* Extracts

Having established that neither Smc/kleisin interface is obligatory for cohesin loading or DNA entrapment in yeast, we addressed whether hinge opening is required. We therefore sought to test the effect of crosslinking Smc1 and Smc3 moieties of the hinge using bi-functional thiol-specific reagents such as BMOE and bBBr. However, this approach cannot be applied to yeast cells for two reasons. Both BMOE and bBBr are lethal to yeast and any reduction of CMs or CDs could be attributable to non-specific toxicity as well as any topological barrier created by the crosslinking. Even if these crosslinkers were not toxic and permitted CM and/or CD formation, it would be impossible to exclude the possibility that any CMs and CDs detected had been created by crosslinking of complexes already associated with chromatin.

**Figure 7:**
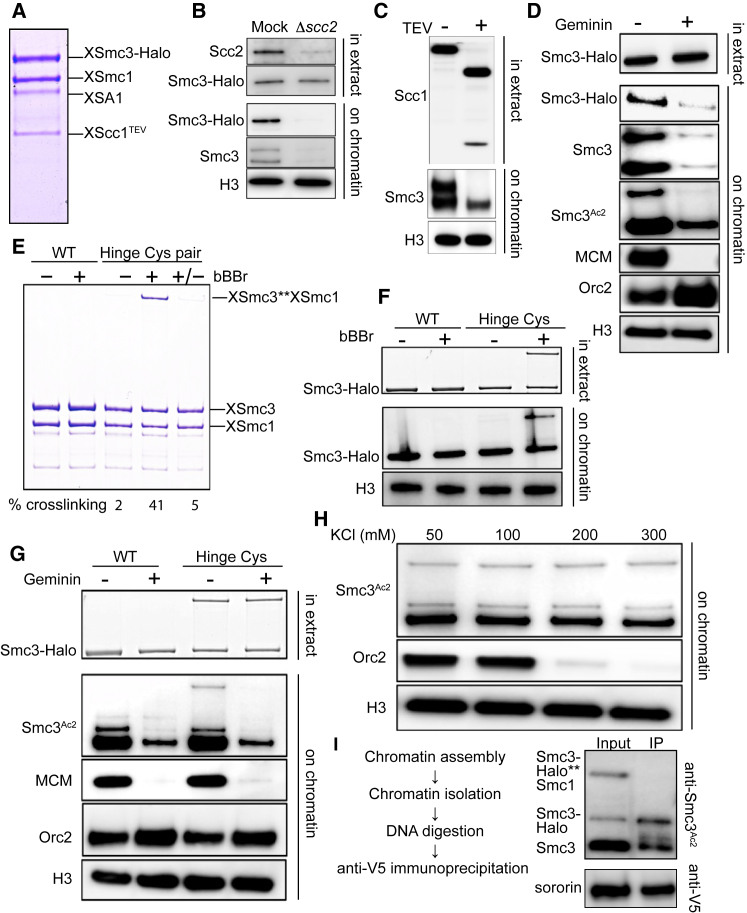
Covalent Closure of Cohesin’s Hinge Interface Fails to Block Loading (A) Coomassie-stained gel showing the *Xenopus* cohesin tetramer purified from baculovirus-infected *Sf-9* cells. (B) Mock- and Scc2-depleted interphase low-speed supernatants (LSS) *Xenopus* egg extracts were supplemented with the recombinant *Xenopus* tetramer and sperm nuclei and incubated at 23°C for 90 min. The isolated chromatin fraction and the soluble extracts were analyzed by western blotting using indicated antibodies. (C) Recombinant *Xenopus* tetramer was treated with TEV protease or buffer for 60 min at 16°C. The reaction was then mixed with LSS interphase *Xenopus* egg extracts and treated as in (B); the chromatin and soluble fractions were analyzed by western blotting using indicated antibodies. (D) LSS interphase extract was treated with either purified recombinant geminin (60 nM) or buffer for 15 min on ice. The extracts were then supplemented with recombinant *Xenopus* tetramer and sperm chromatin and treated as in (A). The chromatin and soluble fractions were analyzed by western blotting using indicated antibodies. (E) Recombinant WT cohesin and cohesin complex containing cysteine substitutions in the hinge domain (Hinge Cys) were treated with DMSO (–), 125 μM bBBr (+), or 125 μM bBBr with 10 mM DTT (+/−). Samples were also supplemented with tetramethylrhodamine (TMR) HaloTag ligand and incubated on ice for 10 min and then run on a 3%–10% gradient gel. The crosslinking efficiency was quantified via TMR fluorescence. (F) WT and hinge substituted *Xenopus* tetramer was treated with DMSO or bBBr on ice for 10 min, and excess crosslinker was then quenched by adding 10 mM DTT. The reactions were then supplemented with interphase extracts, TMR ligand, and sperm chromatin and treated as in (B). The soluble and chromatin fractions were analyzed by TMR fluorescence and indicated antibodies. (G) Crosslinking reactions described in (F) were supplemented with extracts pre-treated with buffer or recombinant geminin and western blots performed as described in (D). (H) Hinge substituted cohesin was crosslinked and supplemented with interphase extracts and 3 ng BAC DNA/μL. After a 90 min incubation, chromatin fractions were isolated, and the chromatin pellets were washed with buffer containing indicated amounts of KCl and analyzed by western blotting. (I) Hinge substituted *Xenopus* tetramer was crosslinked and loaded onto chromatin as in (F). The isolated chromatin pellet washed with buffer containing 300 mM KCl. The pellet was then re-suspended in Xenopus B (XB) buffer supplemented with anti-V5 antibody and benzonase (1 U/μL) and incubated at 12°C overnight. The immunoprecipitates were analyzed by western blot. See also Figure S6.

We therefore studied loading onto chromatin *in vitro* of a *Xenopus* Smc1/Smc3/Scc1/Scc3 (SA1) tetramer purified from insect cells (Figure 7A). To distinguish exogenous and endogenous complexes, the former’s Smc3 subunit was tagged at its C terminus with Halo (Smc3-Halo), while its Scc1 subunit contained three tandem TEV protease cleavage sites. Addition of sperm chromatin to low-speed supernatant (LSS) interphase egg extracts leads to chromatin assembly, cohesin loading, acetylation of Smc3, and eventually DNA replication (Lafont et al., 2010, Takahashi et al., 2004). Like endogenous Smc3, Smc3-Halo association with chromatin dependented on Scc2 (Figure 7B) and was abolished by TEV-induced Scc1 cleavage (Figure 7C). Importantly, inhibition of pre-replication complex assembly by geminin addition greatly reduced association with chromatin as well as acetylation of both versions of Smc3 (Figure 7D) (Takahashi et al., 2008).

To address whether this loading requires opening of the hinge, we produced a version of the complex containing Smc1D566C and Smc3R626C, whose cysteines at the hinge interface can be crosslinked with 40% efficiency using bBBr (Figure 7E). After crosslinking, the reaction was quenched with DTT and the purified complexes added to extracts. Importantly, the crosslinking reaction, which will modify all surface cysteines, did not adversely affect loading of exogenous WT complex. Strikingly, Smc1D566C/Smc3R626C complexes whose hinges had been crosslinked were loaded onto chromatin (Figure 7F) and acetylated (Figure 7G) with similar efficiency to that of uncrosslinked complexes. Furthermore, the crosslinked and acetylated complexes were resistant to 0.3 M KCl, which abolishes most of the Orc2-DNA interaction (Figure 7H). This suggests that complexes whose hinges cannot open are capable of loading onto chromatin in *Xenopus* extracts in a manner that permits their subsequent acetylation by Esco2.

**Figure S6:**
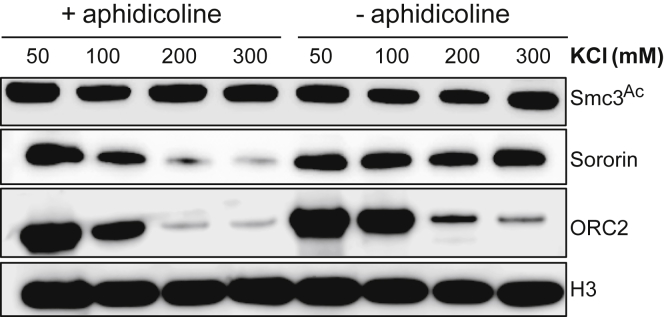
Related to Figure 7 Interphase *Xenopus* egg extract was treated with either DMSO or aphidicholine for 15 min. The extracts were then supplemented with 3 ng BAC DNA/μl. After a 90 min incubation, chromatin fractions were isolated, the chromatin pellets washed with buffer containing indicated amounts of KCl and analyzed by western blotting.

We next asked whether chromatin bound crosslinked complexes were bound to sororin. While Smc3 acetylation is sufficient to counteract releasing activity in yeast, in higher eukaryotes sororin association with acetylated cohesin during S phase is necessary to counteract Wapl activity and to maintain cohesion (Lafont et al., 2010, Nishiyama et al., 2010). Sororin’s association with chromatin becomes salt-resistant following replication (Figure S6). We therefore assembled chromatin using bacterial artificial chromosomes (BACs), which can replicate in egg extract (Aze et al., 2016), and loaded the hinge crosslinked cohesin in interphase extracts supplemented with recombinant sororin. Chromatin pellets were then isolated, subjected to a salt wash and digested by benzonase. Sororin immunoprecipitation (IP) followed by detection of the associated acetylated Smc3 revealed that, while endogenous Smc3 and the uncrosslinked recombinant Smc3 were associated with sororin, hinge crosslinked complexes were not (Figure 7I). If salt-resistant sororin binding reflects cohesion, then hinge crosslinking would appear to abrogate cohesion establishment.

## Discussion

### Re-evaluation of the Ring Model

Elucidation of cohesin’s basic geometry led to the notion that sister DNAs are held together by co-entrapment inside a tripartite ring formed by pairwise interactions among Smc1, Smc3, and Scc1. This is known as the ring model. We describe here the first systematic attempt to test a key prediction of the ring model, namely, that dimeric DNAs catenated by cohesin rings in this manner (CDs) should invariably be found in post-replicative cells that have generated cohesion while individual DNAs catenated by cohesin rings (CMs) should always be formed when cohesin is known to load onto chromosomes.

With the creation of a wide variety of cohesin mutations, this undertaking had become timely. This approach was both powerful and rigorous as only a single counter-example would be sufficient to disprove either hypothesis. Our results reveal a perfect correlation between formation of CDs and cohesion *in vivo*.

In contrast, despite a strong correlation between cohesin loading *in vivo* and CM formation, our approach revealed a counter-example, namely, cohesin complexes with multiple mutations (*smc1DDsmc3AAA*) that reduced the positive charge of the small lumen within the Smc1/3 hinge. *smc1DDsmc3AAA* cohesin loads onto and moves along chromatin as well if not better than WT and yet it largely fails to form CMs. This finding suggests that when cohesin associates with chromatin without forming cohesion it can do so in two ways: one involving strict “topological” entrapment of individual chromatin fibers within cohesin rings (as detected by CMs) and another that does not. It seems implausible to imagine that the non-topological association is an artifact caused uniquely by *smc1DDsmc3AAA*. The most parsimonious explanation is that WT cohesin uses both non-topological and topological modes and that *smc1DDsmc3AAA* can perform the former but not the latter. Though proficient in loading, *smc1DDsmc3AAA* cohesin cannot support sister chromatid cohesion, emphasizing the importance of topological entrapment for this process.

What is the nature of cohesin’s non-topological association with chromatin? One possibility is that it involves entrapment of DNA loops instead of individual DNA segments inside cohesin rings. If loop extrusion extended such loops sufficiently, then the non-topological association would still be topological in nature, though our minichromosome assay would not detect this. Nevertheless, it is equally possible that the non-topological mode does not involve any kind of entrapment of DNA within cohesin rings. It might instead involve association of DNA with Scc3 bound to Scc1 in manner similar to that observed with condensin’s Ycg1 subunit (Kschonsak et al., 2017).

### Functional Interactions between Cohesin Rings

Our demonstration that WT Scc1 enables a version that cannot bind Pds5 (Scc1V137K) to form CDs implies that two or more cohesin rings interact in a functional manner to create cohesive structures. Complementation between different defective *scc1* alleles points to the same conclusion (Eng et al., 2015). If, as our results suggest, cohesion is mediated by entrapment of sister DNAs within individual cohesin rings, it is not obvious why an interaction between rings would be necessary. We cannot at this juncture exclude the possibility that WT cohesin facilitates CD formation by V137K cohesin rings merely because the former hold sister DNAs together. In other words, the suppression of V137K’s cohesion defect need not imply any direct functional interaction between WT and mutant rings.

### Topological Entrapment, but Not Loading, Requires a DNA Entry Gate

If cohesion is mediated by co-entrapment, then the cohesin ring must transiently open up to permit DNA entry. If we assume that there is only a single-entry gate, then it cannot be situated at either of the two Smc-Scc1 interfaces because, as we show here, DNAs still enter cohesin rings containing either Smc3-Scc1 or Scc1-Smc1 fusion proteins. This leaves the Smc1/3 hinge interface.

Unlike the Smc/kleisin interfaces, it is impossible to block hinge opening by making gene fusions, and we therefore addressed the issue using two different approaches. First, it should be possible to inactivate the gate by mutating residues within it. We suggest that the simplest explanation for the phenotype of the *smc1DDsmc3AAA* hinge allele is that it prevents entrapment, either by blocking opening or passage of DNA through it. The second approach involved testing the effect of thiol-specific crosslinks across the Smc1/3 hinge interface of *Xenopus* complexes. Consistent with the *smc1DDsmc3AAA* phenotype, this had no effect on loading but blocked association between chromosomal cohesin and sororin. If sororin association is a mark of cohesive complexes, then it would appear that sealing the hinge interface is sufficient to prevent their formation.

Another important implication of the *smc1DDsmc3AAA* phenotype is that the whole notion of a DNA entry gate being necessary for cohesin loading may be fundamentally misconceived. If loading is not usually accompanied by DNA’s topological entrapment, then there is simply no need for an entry gate.

### The Hinge Is Required for Loading, as well as DNA Entrapment

One of our most unexpected findings is that the hinge has a key role in loading cohesin onto chromosomes as well as DNA entrapment. A remarkable aspect of the loading function is its disruption through substitution by acidic residues of three highly conserved Smc1 lysine residues inside the hinge’s lumen (*smc1DDD*). Because loading is unaffected by mutating two out three residues, all three must have equivalent roles, including one, namely, K650, that unquestionably points inside the hinge’s lumen. Because *smc1DDD* cohesin forms normal rings *in vivo* and is fully active as an ATPase *in vitro*, we suggest that its loading defect arises because it fails to execute an action that normally accompanies the ATP hydrolysis cycle during the loading process. We therefore propose that the three lysines act through their transient exposure to a negatively charged substrate, such as DNA. Thus, a change in the hinge’s conformation may be required for loading as well as DNA entrapment.

An important clue as to the nature of this change is that *smc1K554D K661D* complexes, which behave like WT, are converted to ones that cannot load at all by *smc1K650D* but to ones that can load but not entrap by *smc3R665A K668A R669A*. The implication is that there is something in common between the hinge conformational changes necessary for loading and the process of entrapment. If the latter involves hinge opening, then the former might involve a more modest change that merely exposes Smc1K554 K650 K661 to their substrate. We speculate that, being normally hidden inside the hinge’s lumen, these lysines are only exposed to their substrate (possibly DNA) transiently, at a certain stage of the ATP hydrolysis cycle mediated by Scc2. In other words, the inside surface of the cohesin’s hinge may act as a DNA binding pocket whose access is regulated by its ATPase.

**Figure S7:**
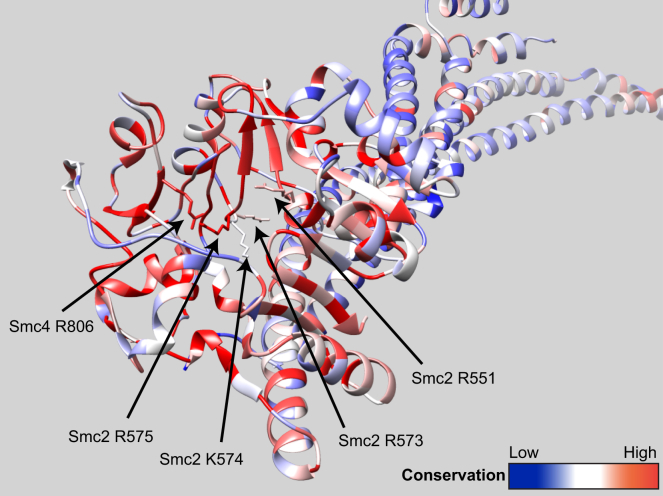
Related to Figures 4 and 5 The conserved positively charged residues that lie inside the lumen of condensin hinge domain are marked in the structure of Smc2-Smc4 hinge domain.

We note that the lumen within condensin’s hinge also contains highly conserved basic residues (Figure S7). One of these corresponds to Smc1K650 (Smc4R806) while the other four are unique to condensin. A role for Smc hinges in the loading and migration may therefore apply to all Hawk-containing Smc/kleisin complexes (Wells et al., 2017). This could conceivably extend to Kite-containing complexes (Palecek and Gruber, 2015). There is a striking similarity between the phenotype caused by *smc1DDD* and that by alterations in the length of Smc coiled coils in *B. subtilis* (Bürmann et al., 2017). Both affect loading and translocation without adversely affecting ATPase activity *in vitro* or indeed association of E1158Q mutation (EQ) complexes with loading sites. Thus, they are both specifically “defective in coupling ATP hydrolysis to essential DNA transactions on the chromosome” (Bürmann et al., 2017). It is therefore conceivable that the event that is disrupted by *smc1DDD* shares features with that disrupted by altering the phase of Smc coiled coils in *B. subtilis*.

If DNAs associate with Scc3 and/or with ATPase heads (Liu et al., 2016) as well as with Smc1/3 hinges, then ATP-driven changes in the relative position of hinges and heads could lie behind cohesin’s ability to move along chromatin. Elucidating the mechanism by which ATP binding/hydrolysis bring about conformational changes in the hinge and coiled coils has the potential to reveal the universal enzymatic principle that organizes chromosomal DNA in most organisms on this planet.

## STAR★Methods

### Key Resources Table

**Table:** 

REAGENT or RESOURCE	SOURCE	IDENTIFIER
**Antibodies**		

Anti-FLAG	Sigma	Cat# F1804
Anti-H3	Rob Klose lab	N/A
Anti-HA	Roche	Cat# 11867423001
Anti-MCM7	SantaCruz	Cat# 47DC141
Anti-MYC	Millipore	Cat# 05-419
Anti-Orc2	Julian Blow lab	N/A
Anti-Scc1/Rad21	Abcam	Cat# ab154769
Anti-Smc3	Bethyl Laboratories	Cat# A300-060A
Anti-Smc3^AC^	Katsu Shirahige lab	N/A
Anti-Sororin	JM Peters lab	N/A
Anti-V5	BioRad	Cat# MCA1360

**Bacterial and Virus Strains**		

*Escherichia coli* Rosetta (DE3) pLysS	Merck	Cat# 70954
MAX Efficiency DH10Bac Competent Cells	ThermoFisher	Cat# 10361012

**Biological Samples**		

*Xenopus* egg extracts	This study	N/A

**Chemicals, Peptides, and Recombinant Proteins**		

Acid-washed glass beads	Sigma	Cat# G8722
Aphidicolin	Sigma	Cat# A0781
ATP α-^32^P	Hartmann Analytic	Cat# SRP-203
Bismaleimidoethane (BMOE)	ThermoFisher	Cat# 22323
Calcium ionophore	Sigma	Cat# A23187
Chorionic gonadotropin	Sigma	Cat# CG10
Complete EDTA free protease inhibitor cocktail	Roche	Cat# 4693132001
Dibromobimane (bBBr)	Sigma	Cat# 34025
Dithiothreitol	Fluka	Cat# BP172
DMSO	Sigma	Cat# D8418
Immobilon Western ECL	Millipore	Cat# WBLKS0500
Indole-3-acetic acid (auxin)	Sigma	Cat# I3750-5G-A
Nocodazole	Sigma	Cat# M1404
PMSF	Sigma	Cat# 329-98-6
Potassium chloride	Sigma	Cat# P5405
Proteinase K	Roche	Cat# 03115836001
RNase A	Roche	Cat# 10109169001
Sodium sulfite	Sigma	Cat# 71988
TMR ligand	Promega	Cat# G8251
Trisodium citrate	Sigma	Cat# W302600
VectaShield with DAPI	Vector Labs	Cat# H-1200
α-factor peptide	CRUK Peptide Synthesis Service	N/A
Human geminin	Costanzo Lab	N/A
*Saccharomyces cerevisiae* cohesin	This study	N/A
*Saccharomyces cerevisiae* cohesin hinge domain	This study	N/A
*Saccharomyces cerevisiae* Scc2	This study	N/A
*Xenopus laevis* cohesin tetramer	This study	N/A

**Critical Commercial Assays**		

AxyPrep Mag PCR Clean up Kit	Appleton Woods	Cat# AX402
ChIP Clean and Concentrator Kit	Zymo Research	Cat# D5205
E-Gel SizeSelect II Agarose Gels, 2%	ThermoFisher	Cat# G661012
EnzChek phosphate assay kit	Invitrogen	Cat# E6646
HiTrap TALON column	GE Healthcare	Cat# 28-9537-67
Library Quantification Kit Ion Torrent Platforms	KAPA Biosystems	Cat# KR0407
Microcon YM-100 columns	Sigma	Cat# Z648094
NEBNext Fast DNA library prep set for Ion Torrent	NEB	Cat# E6270L
NuPAGE 3-8% Tris-Acetate Protein Gels, 1.5 mm, 10-well	ThermoFisher	Cat# EA0378BOX
NuPAGE 4-12% Bis-Tris Protein Gels, 1.0 mm, 10-well	ThermoFisher	Cat# NP0321BOX
Prime-it II Random Primer Labeling Kit	Agilent	Cat# 300385
Protein G dynabeads	ThermoFisher	Cat# 10003D
Slide-a-lyzer dialysis units (3.5kDa)	ThermoFisher	Cat# 66330
StrepTrap HP column	GE Healthcare	Cat# 28-9075-48
Superdex 200 16/60 GL	GE Healthcare	Cat# 17-1069-01
Superose 6 10/300 GL	GE Healthcare	Cat# 17517201
TALON Superflow metal affinity resin	Clontech	Cat# 635670

**Deposited Data**		

GEO accession number	This study	GEO: GSE105005

**Experimental Models: Cell Lines**		

Sf9 cells in Sf-900 II SFM	ThermoFisher	Cat# 11496015

**Experimental Models: Organism/Strains**		

*Xenopus laevis* females	Nasco	LM00535MX
*Xenopus laevis* males	Nasco	LM00715MX
*S. cerevisiae MATa ade2-1 trp1-1 can1-100 leu2-3,112 his3-11,15 ura3 GAL psi+ All following strains are based on this background*	This study	K699
*S. cerevisiae MATa smc1::kanMX4 ura3::smc1(K554D,K661D)-pk12*	Kurze et al., 2011	K15322
*S. cerevisiae Mata smc1::kanMX4 ura3::SMC1-pk12*	Kurze et al., 2011	K15324
*S. cerevisiae Mata smc1::kanMX4 ura3::SMC1(K650D,K661D)-pk12*	Kurze et al., 2011	K15326
*S. cerevisiae Mat a smc1::HIS leu2::smc3(R665A, K668A, K669A)-HA3::LEU2 ura3::smc1(K554D, K661D)-myc9::URA3*	Kurze et al., 2011	K15424
*S. cerevisiae Mat a smc1::HIS leu2::SMC3-HA3::LEU2 ura3::SMC1-myc9::URA3*	Kurze et al., 2011	K15426
*S. cerevisiae MATa/α SMC1-EGFP::HIS3 Mtw1-RFP::KanMX ADE2*	This study	K17660
*S. cerevisiae MATa/α SCC1-EGFP::HIS3 Mtw1-RFP::KanMX ADE2*	This study	K18194
*S. cerevisiae MATa smc1(G22C,K639C)::NatMX4 smc3(E570C,S1043C)::ADE2 scc1(A547C)-PK6::KanMX rad61::hphMX4 leu2::Gal1p-Sic1(9 m)/His3p-Gal1/His3p-Gal2/Gal1p-Gal4 (single copy) 2.3 kb Trp1-ARS1-Cen4 plasmid*	This study	K23451
*S. cerevisiae MATa eco1-1(G211H) smc1(G22C,K639C)::NatMX4 smc3(E570C,S1043C)::ADE2 scc1(A547C)-PK6::KanMX rad61::hphMX4 2.3 kb Trp1-ARS1-Cen4 plasmid*	This study	K23578
*S. cerevisiae MATa eco1-1(G211H) smc1(G22C,K639C)::NatMX4 smc3(E570C,S1043C)::ADE2 scc1(A547C)-PK6::KanMX 2.3 kb Trp1-ARS1-Cen4 plasmid*	This study	K23579
*S. cerevisiae MATa Scc1-PK9::KanMX smc1(G22C,K639C)::NatMX4, smc3(E570C,S1043C)::ADE2 2.3 kb Trp1-ARS1-Cen4 plasmid*	This study	K23644
*S. cerevisiae MATa scc1(A547C)-PK6::KanMX smc3(E570C,S1043C)::ADE2 smc1(G22C,K639C)::NatMX 2.3 kb Trp1-ARS1-Cen4 plasmid*	This study	K23889
*S. cerevisiae MATα smc3(E570C,S1043C)::ADE2 smc1(G22C,K639C)::NatMX leu2::Gal1p-Sic1(9 m)/His3p-Gal1/His3p-Gal2/Gal1p-Gal4 (single copy) 2.3 kb Trp1-ARS1-Cen4 plasmid*	This study	K23890
*S. cerevisiae MATa SCC1-PK9::KanMX smc3(E570C,S1043C)::ADE2 smc1(G22C,K639C)::NatMX leu2::Gal1p-Sic1(9 m)/His3p-Gal1/His3p-Gal2/Gal1p-Gal4 (single copy) 2.3 kb Trp1-ARS1-Cen4 plasmid*	This study	K23971
*S. cerevisiae MATa scc1(A547C)-PK6::KanMX smc3(E570C,S1043C)::ADE2 smc1(G22C,K639C)::NatMX leu2::Gal1p-Sic1(9 m)/His3p-Gal1/His3p-Gal2/Gal1p-Gal4 (single copy) 2.3 kb Trp1-ARS1-Cen4 plasmid*	This study	K23972
*S. cerevisiae MATa scc1(A547C)-PK6::KanMX smc3(E570C,S1043C)::ADE2 smc1(G22C,K639C)::NatMX pds5::HIS3 pds5-101::LEU2 2.3kb Trp1-ARS1-Cen4 plasmid*	This study	K24030
*S. cerevisiae MATa scc1(A547C)-PK6::KanMX smc3(E570C,S1043C)::ADE2 smc1(G22C,K639C)::NatMX cdc4-1::HIS3 2.3 kb Trp1-ARS1-Cen4 plasmid*	This study	K24087
*S. cerevisiae scc1(A547C)::His3MX6 smc3(S1043C,E570C)-PK6::URA3 smc1(G22C,K639C)::NatMX 2.3 kb Trp1-ARS1-Cen4 plasmid*	This study	K24173
*S. cerevisiae scc1(A547C)::His3MX6 smc3(E1155Q,S1043C,E570C)-PK6::URA3 smc1(G22C,K639C)::NatMX 2.3 kb Trp1-ARS1-Cen4 plasmid*	This study	K24174
*S. cerevisiae scc1(A547C)::His3MX6 smc3(K38I,S1043C,E570C)-PK6::URA3 Smc1(G22C,K639C)::Nat 2.3 kb Trp1-ARS1-Cen4 plasmid*	This study	K24175
*S. cerevisiae MATa Smc3(E570C,S1043C)::ADE2 Smc1(G22C,K639C)::NatMX4 URA3::Pscc1-SCC1(A547C)-Pk9 2.3 kb Trp1-ARS1-Cen4 plasmid*	This study	K24205
*S. cerevisiae MATa Scc1(A547C)-pk6::KanMX Smc3(E570C,S1043C)::ADE2 Smc1(G22c,K639C)::NatMX scc2-45::natMX (L545P D575G) 2.3 kb Trp1-ARS1-Cen4 plasmid*	This study	K24267
*S. cerevisiae MATa Scc1(A547C)::His3MX6 URA3::SMC1(G22C,K639C)-PK12 Smc3(E570C,S1043C)::ADE2 2.3 kb Trp1-ARS1-Cen4 plasmid*	This study	K24327
*S. cerevisiae Tetraploid Smc3(E570C,S1043C)::ADE2 x4 Smc1(G22c,K639C)::NatMX x4 Scc1 x3 Scc1(A547C)-PK6::KanMX 2.3 kb Trp1-ARS1-Cen4 plasmid*	This study	K24560
*S. cerevisiae Tetraploid Smc3(E570C,S1043C)::ADE2 x4 Smc1(G22c,K639C)::NatMX x4 Scc1(A547C) x3 Scc1(A547C)-PK6::KanMX 2.3 kb Trp1-ARS1-Cen4 plasmid*	This study	K24561
*S. cerevisiae Mata Smc1(G22C,K639C)::NatMX4 Smc3(E570C,S1043C)::ADE2 leu2::Gal-Scc1(R180E,R268D,A547C)-PK6 2.3 kb Trp1-ARS1-Cen4 plasmid*	This study	K24695
*S. cerevisiae MATa Smc1(G22C,K639C)::NatMX4 Smc3(E570C,S1043C)::ADE2 leu2::Gal-Scc1(R180E,R268D,A547C)-PK6 scc3(K404E)-HA3::HIS3 2.3 kb Trp1-ARS1-Cen4 plasmid*	This study	K24697
*S. cerevisiae MATa Smc1(G22C,K639C)::NatMX4 ura3::Scc1P-Smc3(E570C)-TEV3-Scc1(A547C)-PK9::KanMX (single integrant, fusion linker: (GGGGS)x8+TEV3) 2.3 kb Trp1-ARS1-Cen4 plasmid*	This study	K24838
*S. cerevisiae MATa Smc1(G22C,K639C)::NatMX4 Smc3(E570C,S1043C)::ADE2 Scc1(A547C)-PK6::KanMX rad61::hphMX4 eco1::HIS3 2.3 kb Trp1-ARS1-Cen4 plasmid*	This study	K25287
*S. cerevisiae MATa Scc2-PK6::KANMX6 URA3::smc1(E1158Q)-myc9*	This study	K25646
*S. cerevisiae MATa scc1::NatMX4 smc1::KANMX4 Smc3(E570C,S1043C)::ADE2 Leu2::Scc1p-PK3-Scc1-(GGGGSx13+3xTEV)-Smc1(K649C) 2.3 kb Trp1-ARS1-Cen4 plasmid*	This study	K25696
*S. cerevisiae MATa Smc3(E570C,S1043C)::HIS3MX6 Scc1(A547C)-PK6::KanMX ura3::Smc1(G22C,K639C)-MYC9 2.3 kb Trp1-ARS1-Cen4 plasmid*	This study	K26210
*S. cerevisiae MATa smc3(R665A,K668A,R669A,E570C,S1043C)::HIS3MX6 Scc1(A547C)-PK6::KanMX ura3::smc1(G22C,K554D,K639C,K661D)-Myc9 2.3 kb Trp1-ARS1-Cen4 plasmid*	This study	K26215
*S. cerevisiae MATa Smc1(G22C,K639C)::NatMX4 Smc3(E570C,S1043C)::ADE2 ura3::Pscc1-scc1(A547C,V137K)-PK9 2.3 kb Trp1-ARS1-Cen4 plasmid*	This study	K26413
*S. cerevisiae MATa Smc1(G22C,K639C)::NatMX4 Smc3(E570C,S1043C)::ADE2 scc1(S525N)::His3MX6 (scc1-73) ura3::Pscc1-SCC1(A547C,V137K)-PK92.3 kb Trp1-ARS1-Cen4 plasmid*	This study	K26591
*S. cerevisiae MATa Smc1(G22C,K639C)::NatMX4 Smc3(E570C,S1043C)::ADE2 URA3::Pscc1-SCC1(A547C)-Pk9 scc1(S525N)::His3MX6 (scc1-73) 2.3 kb Trp1-ARS1-Cen4 plasmid*	This study	K26600
*S. cerevisiae MATa Scc1(A547C)::His3MX6 URA3::smc1(G22C,K554D,K639C,K650D,K661D)-PK12*	This study	K26610
*S. cerevisiae MATa smc1(K661D,K554D) Trp1::smc3(R665A,K668A,R669A)-HA3 smc3-3slAA::KanMX ura3::OSTIR1-2-9MYC*	This study	K26611
*S. cerevisiae mata/α ADE2/ADE2 Mtw1-RFP::KanMX/Mtw1-RFP::KanMX smc3(R665A,K668A,R669A)/smc3(R665A,K668A,R669A) ura3::smc1(K554D,K661D)-EGFP/ura3::smc1(K554D,K661D)-EGFP*	This study	K26700
*S. cerevisiae MATa ura3::smc1(K554D,K650D,K661D)-PK12*	This study	K26756
*S. cerevisiae MATa, ura3::Smc1(K554D, K650D, K661D)-PK12::URA3*	This study	K26756
*S. cerevisiae Mat a smc3(R665A, K668A, R669A) ura3::smc1(K554D, K650D, K661D)-PK12::URA3*	This study	K26757
*S. cerevisiae Mat a SMC3-3slAA::KAN ura3::OSTIR1-2-9MYC::URA3*	This study	K26767
*S. cerevisiae Mata SMC3-3sIAA::KanMX ura3::OSTIR1-2-9MYC trp1::SMC3-HA*	This study	K26797
*S. cerevisiae MATa ura3::Smc1-Myc9 Scc2-PK6::KANMX6*	This study	K26839
*S. cerevisiae MATa ura3::smc1(K554D,K650D,K661D,E1158Q)-Myc9 Scc2-PK6::KANMX6*	This study	K26840

**Recombinant DNA**		

pAceBac1 6His-GFP-(Δ1-132)Scc2-Strep	This study	N/A
pAceBac1 6His-SMC1 SMC3	Petela et al., 2017	N/A
pAceBac1 6His-SMC1(K554D, K650D, K661D) SMC3	This study	N/A
pAceBac1 6His-SMC1(K554D, K661D) SMC3(R665A,K668A,R669A)	This study	N/A
pET28 SMC3 hinge (494-705, E570C), SMC1 hinge (486-696, K554D, K650D, K661D)-His6	This study	N/A
pET28 SMC3 hinge (494-705, E570C), SMC1 hinge (486-696, K639C)-His6	Haering et al., 2008	N/A
pET28 SMC3 hinge (494-705, E570C), SMC1 hinge (486-696)-His6	Haering et al., 2008	N/A
piDC SCC1-twinstrep SCC3	Petela et al., 2017	N/A
pFastBac Dual XSCC1-TEV XSA1	This study	N/A
pFastBac Dual XSMC3-HALO XSMC1	This study	N/A
pMAL MBP-SMC1 hinge (503-681, K639C)-His6	This study	N/A

**Software and Algorithms**		

Galaxy platform	Giardine et al., 2005	https://usegalaxy.org
FastQC	Galaxy tool version 1.0.0	https://usegalaxy.org
Trim sequences	Galaxy tool version 1.0.0	https://usegalaxy.org
Filter FASTQ	Galaxy tool version 1.0.0	https://usegalaxy.org
Bowtie2	Langmead and Salzberg, 2012; Galaxy tool version 0.2	https://usegalaxy.org
Bam to BigWig	Galaxy tool version 0.1.0	https://usegalaxy.org
Samtools	Li et al., 2009	http://samtools.sourceforge.net/
IGB browser	Nicol, Helt, Blanchard, Raja, & Loraine, 2009	http://bioviz.org/igb/
Filter SAM or BAM	Li et al., 2009 Galaxy tool version 1.1.0	https://usegalaxy.org
chr_position.py	This study	https://github.com/naomipetela/nasmythlab-ngs
filter.py	This study	https://github.com/naomipetela/nasmythlab-ngs
bcftools call	Li et al., 2009	N/A

### Contact for Reagent and Resource Sharing

Further information and requests for resources and reagents should be directed to and will be fulfilled by the Lead Contact, Kim A. Nasmyth (ashley.nasmyth@bioch.ox.ac.uk).

### Experimental Model and Subject Details

#### Yeast cell culture

All strains are derivatives of W303 (K699). Strain numbers and relevant genotypes of the strains used are listed in the Key Resource Table. Cells were cultured at 25°C in YEP medium with 2% glucose unless stated otherwise. To arrest the cells in G1, α-factor was added to a final concentration of 2 mg/L, every 30 min for 2.5 h. Cells were released from G1 arrest by filtration wherein cells were captured on 1.2 μm filtration paper (Whatman^®^ GE Healthcare), washed with 1 L YEPD and resuspended in the appropriate fresh media. To inactivate temperature sensitive alleles, fresh media was pre-warmed prior to filtration (Aquatron, Infors HT).

To arrest cells in G2, nocodazole (Sigma) was added to the fresh media to a final concentration of 10 μg/mL and cells were incubated until the synchronization was achieved (> 95% large-budded cells).

Cells were arrested in late G1 by galactose-induced overexpression of a non-degradable mutant of the Sic1 protein (mutation of 9 residues phosphorylated by Cdk1). To achieve this, cells were grown in YEP supplemented with 2% raffinose and arrested in G1 as described above. 1 h before release from G1 arrest, galactose was added to 2% of the final concentration. Cells were released into YEPD as described above, and incubated for 60 min at 25°C.

For auxin induced degradation of proteins, cells were arrested in G1 as above and 1 h prior to release auxin (indole-3-acetic acid sodium salt; Sigma) was added to a final concentration of 1 mM. Cells were released from G1 arrest into YEPD medium containing 1 mM auxin and 10 μg/mL nocodazole.

#### *Xenopus* laevis frogs

Eggs derived from *Xenopus laevis* frogs were used as experimental model system. Collection of eggs from the female frogs was performed in a non-invasive way following chorionic gonadotropin (Sigma, CG10) injections. Occasional surgical procedures were performed on the male frogs to harvest sperm nuclei. Experimental protocols were approved by IFOM Animal Welfare committee and the Italian Ministry of Health. The number of animals used was kept to a minimum and was calculated taking into account the number eggs required to obtain the cytoplasmic extract needed for the experiments described.

The animals were kept in highly regulated and monitored conditions with room and water temperature at 19°C. Basic husbandry requirements were provided by the IFOM *Xenopus* facility.

### Method Details

#### *In vivo* chemical crosslinking

Strains were grown in YEPD at 25°C to OD_600nm_ = 0.5-0.6. 12 OD units were washed in ice-cold PBS and re-suspended in 1 mL ice-cold PBS. The suspensions were then split into 2 × 500 μL and 20.8 μL BMOE (stock: 125 mM in DMSO, 5 mM final) or DMSO was added for 6 min on ice. Cells were washed with 2 × 2 mL ice-cold PBS containing 5 mM DTT, resuspended in 500 μL lysis buffer (25 mM HEPES pH 8.0, 50 mM KCl, 50 mM MgSO_4_, 10 mM trisodium citrate, 25 mM sodium sulfite, 0.25% triton-X, freshly supplemented with Roche Complete Protease Inhibitors (2X) and PMSF (1 mM), lysed in a FastPrep-24 (MP Biomedicals) for 3 × 1 min at 6.5 m/s with 500 μL of acid-washed glass beads (425-600 μm, Sigma) and lysates cleared (5 min, 12 k*g*). Protein concentrations were adjusted after Bradford assay and cohesin immuno-precipitated using anti-PK antibody (AbD Serotec, 1 h, 4°C) and protein G dynabeads (1 h, 4°C, with rotation). Beads were washed with 2 × 1 mL lysis buffer, resuspended in 50 μL 2x sample buffer, incubated at 95°C for 5 min and the supernatant loaded onto a either 3%–8% Tris-acetate or 4%–12% Bis-Tris gradient gels (Life Technologies).

#### Minichromosome IP

Strains containing a 2.3 kb circular minichromosome harboring the *TRP1* gene were grown overnight in –TRP medium at 25°C and sub-cultured in YEPD medium for exponential growth (OD_600nm_ = 0.6). 30 OD units were washed in ice-cold PBS and processed for *in vivo* crosslinking as described above with the following modification: after cohesin immuno-precipitation protein G dynabeads were washed with 2 × 1 mL lysis buffer, resuspended in 30 μL 1% SDS with DNA loading dye, incubated at 65°C for 4 min and the supernatant run on a 0.8% agarose gel containing ethidium bromide (1.4 V/cm, 22h, 4°C). After Southern blotting using alkaline transfer, bands were detected using a 32-P labeled TRP1 probe.

#### Calibrated ChIP-sequencing

Cells were grown exponentially to OD_600_ = 0.5 and the required cell cycle stage where necessary. 15 OD_600nm_ units of *S. cerevisiae* cells were then mixed with 5 OD_600nm_ units of *C. glabrata* to a total volume of 45 mL and fixed with 4 mL of fixative (50 mM Tris-HCl, pH 8.0; 100 mM NaCl; 0.5 mM EGTA; 1 mM EDTA; 30% (v/v) formaldehyde) for 30 min at room temperature (RT) with rotation.

The fixative was quenched with 2 mL of 2.5 M glycine (RT, 5 min with rotation). The cells were then harvested by centrifugation at 3,500 rpm for 3 min and washed with ice-cold PBS. The cells were then resuspended in 300 μL of ChIP lysis buffer (50 mM HEPES-KOH, pH 8.0; 140 mM NaCl; 1 mM EDTA; 1% (v/v) Triton X-100; 0.1% (w/v) sodium deoxycholate; 1 mM PMSF; 2X Complete protease inhibitor cocktail (Roche)) and an equal amount of acid-washed glass beads (425-600 μm, Sigma) added before cells were lysed using a FastPrep^®^-24 benchtop homogenizer (M.P. Biomedicals) at 4°C (3 × 60 s at 6.5 m/s or until > 90% of the cells were lysed as confirmed by microscopy).

The soluble fraction was isolated by centrifugation at 2,000 rpm for 3 min then sonicated using a bioruptor (Diagenode) for 30 min in bursts of 30 s on/30 s off at high level in a 4°C water bath to produce sheared chromatin with a size range of 200-1,000 bp. After sonication the samples were centrifuged at 13,200 rpm at 4°C for 20 min and the supernatant was transferred into 700 μL of ChIP lysis buffer. 30 μL of protein G Dynabeads (Invitrogen) were added and the samples were pre-cleared for 1 h at 4°C. 80 μL of the supernatant was removed (termed ‘whole cell extract [WCE] sample’) and 5 μg of antibody (anti-PK (Bio-Rad) or anti-HA (Roche)) was added to the remaining supernatant which was then incubated overnight at 4°C. 50 μL of protein G Dynabeads were then added and incubated at 4°C for 2 h before washing 2x with ChIP lysis buffer, 3x with high salt ChIP lysis buffer (50 mM HEPES-KOH, pH 8.0; 500 mM NaCl; 1 mM EDTA; 1% (v/v) Triton X-100; 0.1% (w/v) sodium deoxycholate; 1 mM PMSF), 2x with ChIP wash buffer (10 mM Tris-HCl, pH 8.0; 0.25 M LiCl; 0.5% NP-40; 0.5% sodium deoxycholate; 1 mM EDTA; 1 mM PMSF) and 1x with TE pH7.5. The immunoprecipitated chromatin was then eluted by incubation in 120 μL TES buffer (50 mM Tris-HCl, pH 8.0; 10 mM EDTA; 1% SDS) for 15 min at 65°C and the collected supernatant termed ‘IP sample’. The WCE samples were mixed with 40 μL of TES3 buffer (50 mM Tris-HCl, pH 8.0; 10 mM EDTA; 3% SDS) and all samples were de-crosslinked by incubation at 65°C overnight. RNA was degraded by incubation with 2 μL RNase A (10 mg/mL; Roche) for 1 h at 37°C and protein was removed by incubation with 10 μL of proteinase K (18 mg/mL; Roche) for 2 h at 65°C. DNA was purified using ChIP DNA Clean and Concentrator kit (Zymo Research).

#### Preparation of sequencing libraries

Sequencing libraries were prepared using NEBNext^®^ Fast DNA Library Prep Set for Ion Torrent Kit (New England Biolabs) according to the manufacturer’s instructions. Briefly, 10-100 ng of fragmented DNA was converted to blunt ends by end repair before ligation of the Ion Xpress Barcode Adaptors. Fragments of 300 bp were then selected using E-Gel^®^ SizeSelect 2% agarose gels (Life Technologies) and amplified with 6-8 PCR cycles. The DNA concentration was then determined by qPCR using Ion Torrent DNA standards (Kapa Biosystems) as a reference. 12-16 libraries with different barcodes could then be pooled together to a final concentration of 350 pM and loaded onto the Ion PI V3 Chip (Life Technologies) using the Ion Chef (Life Technologies). Sequencing was then completed on the Ion Torrent Proton (Life Technologies), typically producing 6-10 million reads per library with an average read length of 190 bp.

#### Data analysis, alignment, and production of BigWigs

Unless otherwise specified, data analysis was performed on the Galaxy platform (Giardine et al., 2005). Quality of reads was assessed using FastQC (Galaxy tool version 1.0.0) and trimmed as required using ‘trim sequences’ (Galaxy tool version 1.0.0). Generally, this involved removing the first 10 bases and any bases after the 200^th^, but trimming more or fewer bases may be required to ensure the removal of kmers and that the per-base sequence content is equal across the reads. Reads shorter than 50 bp were removed using Filter FASTQ (Galaxy tool version 1.0.0, minimum size: 50, maximum size: 0, minimum quality: 0, maximum quality: 0, maximum number of bases allowed outside of quality range: 0, paired end data: false) and the remaining reads aligned to the necessary genome(s) using Bowtie2 (Galaxy tool version 0.2) with the default (–sensitive) parameters (mate paired: single-end, write unaligned reads to separate file: true, reference genome: SacCer3 or CanGla, specify read group: false, parameter settings: full parameter list, type of alignment: end to end, preset option: sensitive, disallow gaps within *n-*positions of read: 4, trim *n*-bases from 5′ of each read: 0, number of reads to be aligned: 0, strand directions: both, log mapping time: false).

To generate alignments of reads that uniquely align to the *S. cerevisiae* genome, the reads were first aligned to the *C. glabrata* (CBS138, genolevures) genome with the unaligned reads saved as a separate file. These reads that could not be aligned to the *C. glabrata* genome were then aligned to the *S. cerevisiae* (sacCer3, SGD) genome and the resulting BAM file converted to BigWig (Galaxy tool version 0.1.0) for visualization. Similarly, this process was done with the order of genomes reversed to produce alignments of reads that uniquely align to *C. glabrata*.

#### Visualization of ChIP-seq profiles

The resulting BigWigs were visualized using the IGB browser (Nicol et al., 2009). To normalize the data to show quantitative ChIP signal the track was multiplied by the samples’ occupancy ratio (OR) and normalized to 1 million reads using the graph multiply function. In order to calculate the average occupancy at each base pair up to 60 kb around all 16 centromeres, the BAM file that contains reads uniquely aligning to *S. cerevisiae* was separated into files for each chromosome using ‘Filter SAM or BAM’ (Galaxy tool version 1.1.0). A pileup of each chromosome was then obtained using samtools mpileup (Galaxy tool version 0.0.1) (source for reference list: locally cached, reference genome: SacCer3, genotype likelihood computation: false, advanced options: basic). These files were then amended using our own script (chr_position.py) to assign all unrepresented genome positions a value of 0. Each pileup was then filtered using another in-house script (filter.py) to obtain the number of reads at each base pair within up to 60 kb intervals either side of the centromeric CDEIII elements of each chromosome. The number of reads covering each site as one successively moves away from these CDEIII elements could then be averaged across all 16 chromosomes and calibrated by multiplying by the samples’ OR and normalizing to 1 million reads.

#### Live-cell imaging

Exponentially growing cells were placed on 2.5% agarose pads made of synthetic complete medium containing glucose. Live cell imaging was performed under a spinning disk confocal system (PerkinElmer UltraVIEW) with an EMCCD camera (Hamamatsu) mounted on an Olympus IX81 microscope with Olympus 100x 1.35N.A. objectives. Image acquisition was done at 25°C. Seventeen to twenty-one Z stacking images were acquired and image deconvolution was done by using Volocity software with 7 iterations and 95% confidence. Fresh samples were prepared every 10 min.

#### Chromosome spreads

Chromosome spreads were done according to a protocol described (Shen and Skibbens, 2017): Cells were fixed with 4% paraformaldehyde for 90 min at 25°C. Cells were washed 3 times with distilled water and resuspended in spheroplast buffer (1M sorbitol, 20 mM KPO4, pH7.4), then spheroplasted by adding β-mercaptoethanol (1/50 volume) and zymolyase T100 (stock: 10 mg/mL; 1/100 volume) and incubating for 1 h at 25°C. Spheroplasts were pelleted and resuspended in 1.5 pellet volume of spheroplast buffer with 0.5% Triton X-100. 10 μL of the cell suspension were added to each well on poly-L-ysine coated slides, set at room temperature for 10 min. After removing the liquid from the wells by gentle pipetting 20 μL of 0.5% SDS was added to each well and set for 10 min at room temperature. After gentle aspiration the slides were air-dried. The attached spheroplasts were then dehydrated by immersing the slides in fresh methanol/acetic acid (3:1) for 5 min at room temperature. Slides were stored at 4°C until completely dry, then treated with RNase A (100 μg/ml) in 2xSSC buffer (0.3 M NaCl, 30 mM sodium Citrate, pH7.0) for 1 h at 37°C. Slides were washed 4 times in fresh 2xSSC (2 min/per wash), then subjected to a series of cold (−20°C) ethanol washes (start with 70%, followed by 80%, 95% ethanol washes, 2 min/per wash), and air-dried. Slides were pre-warmed to 37°C, then put into denaturing solution (70% formamide, 2xSSC) at 72°C for 2 min, and immediately subjected to a series of cold (−20°C) ethanol washes (start with 70%, followed by 80%, 90%, 100% ethanol washes, 1 min/per wash), and air-dried. Slides were mounted with VectaShield mounting medium (Vector Laboratories) and observed under a Zeiss LSM780 confocal microscope equipped with a 100x Plan-Apochromat 1.4NA oil immersion objective lens (Carl Zeiss).

#### Protein purification

##### Hinge domains

*E. coli* Rosetta (DE3) pLysS (Stratagene) were transformed with pET vectors expressing the cohesin hinge domains. The expression was induced at OD_600_ = 0.6 with 1 mM IPTG at 18°C overnight. The cells were pelleted and re-suspendd with TAP buffer (50 mM Tris–HCl, 250 mM NaCl, 1 mM β-mercaptoethanol, pH 7.5), freshly supplemented with EDTA-free protease inhibitor cocktail tablets (Roche). After re-suspension, the cells were lysed with the French press (Constant Systems) at 18 kPsi followed by 1 min sonication at 80% AMPL (Sonics Vibra-Cell). The cell lysate was cleared by centrifugation at 80,000 g for 30 min at 4°C (Beckman Coulter, rotor JLA-16.250). The cleared lysate was incubated with Talon Superflow beads (Clontech) for 2 h at 4°C. Beads were washed 3 times with TAP buffer containing 10 mM imidazole. Proteins were eluted in TAP buffer with 500 mM imidazole and loaded onto a Superdex 200 16/60 chromatography column (GE Healthcare) equilibrated with TAP buffer (50 mM Tris–HCl, 100 mM NaCl, 1 mM β-mercaptoethanol, pH 7.5). Peak fractions were collected and concentrated using Vivaspin columns (Sartorius Stedim Biotech).

##### *Xenopus* proteins

*Xenopus* Smc1 and Smc3 genes were cloned into pFastBac Dual vector (Invitrogen). A C-Terminal tag consisting 2xFLAG and HALO was fused to XSmc3. XSA1 and XRad21 genes were cloned into pFastBac Dual vector. XRad21 was cloned with His8 tag in the C terminus. 3xTEV sites were introduced into a proline-rich region in XRad21 after P465. Baculoviruses for protein expression in Sf9 cells were generated according to the Bac-to-Bac baculovirus expression system protocol (Invitrogen/Thermo Scientific).

Approximately 72 h after baculovirus addition, insect cells were harvested, washed in PBS, frozen in liquid nitrogen and stored at −80°C. All subsequent steps were performed on ice or at 4°C. Cells were lysed by thawing and dounce homogenizing in buffer A500 (25 mM KH_2_PO_4_ pH 7.5, 500 mM KCl, 5% v/v glycerol, 2 mM MgCl_2_), 20 mM β-mercaptoethanol, 0.05% v/v Tween-20, 0.5 mg/ mL PMSF and complete protease inhibitor (Roche). After lysis, an equal volume of buffer A0 (buffer A500 lacking KCl) was added to the lysate and centrifuged at 75,000*g* for 40 min. The clarified lysate was filtered through a 0.45 μm filter and cohesin purified in the AKTA system using a 5 mL (HisTrap) TALON column (VWR). The column was washed with 20 column volumes of buffer B (20 mM Tris PH7.5, 250 mM NaCl, 5% glycerol) and eluted over a linear gradient of 0-500 mM imidazole. The peak fractions containing the cohesin tetramer were pooled and incubated with anti-FLAG-M2 resin (Sigma) for 3 h at 4°C. The beads were pelleted and washed with EB buffer (100 mM KCl, 2.5 mM MgCl_2_ and 50 mM HEPES-KOH pH 7.5) and eluted in EB buffer with FLAG peptide containing 10% glycerol and 5 mM DTT. The eluted protein was passed through a Superose6 column (GE Healthcare) equilibrated with EB buffer containing 10% glycerol. Peak fractions were collected and stored in aliquots at −80°C.

#### *Xenopus* extracts and chromatin isolation

S phase extract capable of performing a single round of replication was prepared as previously described (Aze et al., 2016). Briefly, *Xenopus* eggs were collected in MMR buffer (5 mM K-HEPES pH7.5, 100 mM NaCl, 0.5 mM KCl, 0.25 mM MgSO_4_, 0.5 mM CaCl_2_, 25 μM EDTA) from chorionic gonadotropin injected female frogs. The eggs were de-jellied in 10 mM Tris pH8.0, 110 mM NaCl and 5 mM DTT and rinsed three times in MMR. De-jellied eggs were released in interphase in presence of 5 μM calcium ionophore (Sigma) for 5-6 min, washed three times with MMR and rinsed twice in ice-cold S-buffer (50 mM K-HEPES pH7.5, 50 mM KCl, 2.5 mM MgCl_2_, 250 mM sucrose, 2 mM β-mercaptoethanol). Activated eggs were then packed by centrifugation at 1,200 rpm for one minute and the excess of buffer was discarded. Eggs were crushed at 13,000 rpm for 12 min at 4°C. The crude extract was collected and centrifuged at 70,000 rpm for 12 min at 4°C in a TLA100 rotor (Beckman). The interphase extract was obtained by collecting and mixing the cleared cytoplasmic fraction with the nuclear membranes. For sperm nuclei preparation 4 testes were removed from 2 male frogs and placed in Petri dishes containing 10 mL EB buffer (50 mM KCl, 50 mM HEPES KOH pH7.6, 5 mM MgCl_2_, 2 mM DTT). Testes were finely chopped with razor blade. The material was then transferred to a 15 mL Falcon tube and spun at 2,000 x g, in a swinging bucket rotor for 5 min at 4°C. The pellet was resuspended in a total volume of 2 mL of room temperature SuNaSp buffer (0.25 M sucrose, 75 mM NaCl, 0.5 mM spermidine, 0.15 mM spermine). To remove membranes 100 μL of 2 mg/ml lysolecithin (Sigma) were added and incubated for 10 min at room temperature. Reaction was stopped by adding 3% BSA (Sigma). The pellet was resuspended again in 2 mL EB and spun at 2,000 g for 5 min at 4°C. The final pellet was resuspended in 400 μL EB + 30% glycerol. For BACs preparation RP11-1151L10 BAC was purchased from http://bacpac.chori.org/home.htm. The BAC DNA was isolated from bacteria using QIAGEN Plasmid Maxi Kit. The DNA was resuspended in a solution of 50% CsCl supplemented with 12.5 ng/μl of ethidum bromide and centrifuged for 20 h at 60,000 rpm in a 70.1 Ti rotor (Beckman). Formation of a continuous CsCl gradient allowed the precise recovering of the supercoiled DNA from the nicked, linear or broken DNA. Removal of ethidium bromide from DNA was performed using butanol and the DNA was finally dialyzed overnight in TE buffer (10mM Tris HCl pH8.0, 1mM EDTA). BACs DNA (3 to 10 ng/μl) was added to egg extracts and incubated 90 min. Where necessary, geminin was used at a concentration of 60 nM.

To isolate LSS extract assembled chromatin, samples were diluted in ten volumes of EB buffer (100 mM KCl, 2.5 mM MgCl_2_ and 50 mM HEPES-KOH pH 7.5) containing 0.25% Nonidet P-40 and centrifuged through a 30% sucrose (in EB) layer at 10,000 rpm for 5 min at 4°C using a HB-6 rotor (Sorvall), washed twice with 500 μL EB buffer and centrifuged at 10,000 rpm for 1 min. The pellet was resuspended in Laemmli loading buffer and the proteins resolved by either 4%–15%, 7.5% or 10% SDS-PAGE and analyzed by western blotting with specific antibodies as indicated.

#### Immuno-depletion of extracts

Protein A Dynabeads were pre-incubated overnight at 4°C with anti-Scc2 rabbit antiserum or pre-immune rabbit serum. To immuno-deplete XScc2 from the extracts, 0.5 mL extracts were incubated with antibody-bound Dynabeads for 1 h at 25°C. To fully deplete Scc2 four rounds of depletion were required.

#### ATPase assay

ATPase activity was measured by using the EnzChek phosphate assay kit (Invitrogen) by following the protocol as provided. Cohesin tetramer (Smc1, Smc3, Scc1 and Scc3; final concentration: 50 nM, final NaCl concentration: 50 mM) was added together with a 40 bp long double stranded DNA (700 nM). The reaction was started with addition of ATP to a final concentration of 1.3 mM (final reaction volume: 150 μl). After completion, a fraction of each reaction was run on SDS-PAGE and the gel stained with Coomassie brilliant blue in order to test that the complexes were intact throughout the experiment and that equal amounts were used when testing various mutants and conditions.

### Quantification and Statistical Analysis

#### Southern Blotting

After hybridization, Southern blots were exposed to phosphorimager screens (Fuji) and scanned with an FLA7000 scanner (Fuji). The band intensities were quantified using AIDA image analyzer (version 4.50, Raytest). Intensity of each band was calculated as a percentage of total pixel intensity of the lane. At least three biological replicates were performed for each experiment, means and standard deviations are presented in the figures and figure legends.

#### ATPase assay

ATPase activity was measured by recording absorption at 360 nm every 30 s for 90 min using a PHERAstar FS. ΔΑU at 360 nm was translated to P_i_ release using an equation derived by a standard curve of KH_2_PO_4_ (EnzChek kit). Rates were calculated from the slope of the linear phase (first 10 min). At least two independent biological experiments were performed for each experiment, means and standard deviations are reported for every experiment.

#### rDNA morphology

For each condition, a minimum of 100 cells was scored from 3 biological replicates. Means and standard deviations are reported for each condition.

### Data and Software Availability

#### Scripts

All scripts written for this analysis method are available to download from https://github.com/naomipetela/nasmythlab-ngs.

*Chr_position.py* takes mpileups for *S. cerevisiae* chromosomes and fills in gaps, with each position in the chromosome added given a read depth of 0.

*Filter60.py* reads the files produced by Chr_position.py and takes the read depth for all positions 60 kb either side of the CDEIII for all chromosomes, produces an average for each position and multiples it by the OR. The OR should be derived from the reads aligned in the appropriate bam files (Hu et al., 2015).

#### Calibrated ChIP-seq data

The accession number for the calibrated ChIP-seq data (raw and analyzed data) reported in this paper is GEO: GSE105005.
